# Exosomes as Novel Regulators of Adult Neurogenic Niches

**DOI:** 10.3389/fncel.2015.00501

**Published:** 2016-01-19

**Authors:** Luis Federico Bátiz, Maite A. Castro, Patricia V. Burgos, Zahady D. Velásquez, Rosa I. Muñoz, Carlos A. Lafourcade, Paulina Troncoso-Escudero, Ursula Wyneken

**Affiliations:** ^1^Center for Interdisciplinary Studies on the Nervous System (CISNe), Universidad Austral de ChileValdivia, Chile; ^2^Program for Cell Biology and Microscopy, Universidad Austral de ChileValdivia, Chile; ^3^Instituto de Anatomía, Histología y Patología, Facultad de Medicina, Universidad Austral de ChileValdivia, Chile; ^4^Instituto de Bioquímica y Microbiología, Facultad de Ciencias, Universidad Austral de ChileValdivia, Chile; ^5^Instituto de Fisiología, Facultad de Medicina, Universidad Austral de ChileValdivia, Chile; ^6^Laboratorio de Neurociencias, Facultad de Medicina, Universidad de Los AndesSantiago, Chile

**Keywords:** exosomes, adult neurogenesis, niche, stem cells, extracellular vesicles, psychiatric disorders, neurological disorders, biomarkers

## Abstract

Adult neurogenesis has been convincingly demonstrated in two regions of the mammalian brain: the sub-granular zone (SGZ) of the dentate gyrus (DG) in the hippocampus, and the sub-ventricular zone (SVZ) of the lateral ventricles (LV). SGZ newborn neurons are destined to the granular cell layer (GCL) of the DG, while new neurons from the SVZ neurons migrate rostrally into the olfactory bulb (OB). The process of adult neurogenesis persists throughout life and is supported by a pool of neural stem cells (NSCs), which reside in a unique and specialized microenvironment known as “neurogenic niche”. Neurogenic niches are structured by a complex organization of different cell types, including the NSC-neuron lineage, glial cells and vascular cells. Thus, cell-to-cell communication plays a key role in the dynamic modulation of homeostasis and plasticity of the adult neurogenic process. Specific cell-cell contacts and extracellular signals originated locally provide the necessary support and regulate the balance between self-renewal and differentiation of NSCs. Furthermore, extracellular signals originated at distant locations, including other brain regions or systemic organs, may reach the niche through the cerebrospinal fluid (CSF) or the vasculature and influence its nature. The role of several secreted molecules, such as cytokines, growth factors, neurotransmitters, and hormones, in the biology of adult NSCs, has been systematically addressed. Interestingly, in addition to these well-recognized signals, a novel type of intercellular messengers has been identified recently: the extracellular vesicles (EVs). EVs, and particularly exosomes, are implicated in the transfer of mRNAs, microRNAs (miRNAs), proteins and lipids between cells and thus are able to modify the function of recipient cells. Exosomes appear to play a significant role in different stem cell niches such as the mesenchymal stem cell niche, cancer stem cell niche and pre-metastatic niche; however, their roles in adult neurogenic niches remain virtually unexplored. This review focuses on the current knowledge regarding the functional relationship between cellular and extracellular components of the adult SVZ and SGZ neurogenic niches, and the growing evidence that supports the potential role of exosomes in the physiology and pathology of adult neurogenesis.

## Introduction

It was long believed that mammalian neurogenesis, i.e., the process of generating functional neurons from neural precursors, occurred only during embryonic and perinatal stages (Ming and Song, 2005). Altman’s pioneering studies in the 1960’s provided the first histological evidence for the presence of newborn neurons in the adult brain of rodents (Altman and Das, 1965, 1966; Altman, 1969). However, the fact that young neurons are continuously incorporated into the adult brain circuitry was not widely accepted until the mid-1990’s (Gross, 2000; Kaplan, 2001). Advances in immunohistological techniques and particularly the introduction of bromodeoxyuridine (BrdU), a thymidine analog that can be incorporated *in vivo*, allowed to recognize proliferative populations of cells within the central nervous system (CNS) and to identify the destination of adult-born neurons. To date it is well known that neural stem cells (NSCs) reside in the brain of most adult mammals, including humans, and that neurogenesis does occur throughout life (Eriksson et al., 1998; Gage, 2000; Lie et al., 2004; Abrous et al., 2005; Ming and Song, 2005; Lledo et al., 2006; Merkle and Alvarez-Buylla, 2006; Bergmann et al., 2015; Kempermann et al., 2015). Interestingly, regions harboring active adult neurogenesis are located in discrete but specific areas of the brain. These areas, known as “neurogenic niches”, are composed of different cell types, specific cell-cell contacts, and particular extracellular cues originated both locally and distantly. Thus, the function of the different cellular and molecular components of the niche supports the physiology of NSCs, balancing quiescence with proliferation, and regulating cell differentiation (Conover and Notti, 2008).

In this context, not only the cytoarchitectonic organization of the niche but also the ways of communication between the cellular components, are critical to understand the adult neurogenic process under both physiological and pathological conditions. Cells communicate reciprocally with other cells by (i) intercellular contacts, and (ii) secreted molecules, such as growth factors, cytokines, hormones, etc. (paracrine or endocrine communication). However, a novel way of cell-to-cell communication mediated by extracellular vesicles (EVs) has attracted the attention of several researchers in different fields. EVs, such as exosomes, carry a specific cargo of proteins, lipids and nucleic acids and are currently consider one of the most complex and physiologically relevant messengers between cells. This review focuses on the cellular components of the adult neurogenic niches, the mechanisms involved in intercellular communication, and the potential role of exosomes as regulators of the neurogenic process, and as mediators and novel biomarkers of neuropsychiatric and neurological disorders associated with defective adult neurogenesis.

## Adult Neurogenic Niches

**Figure 1:**
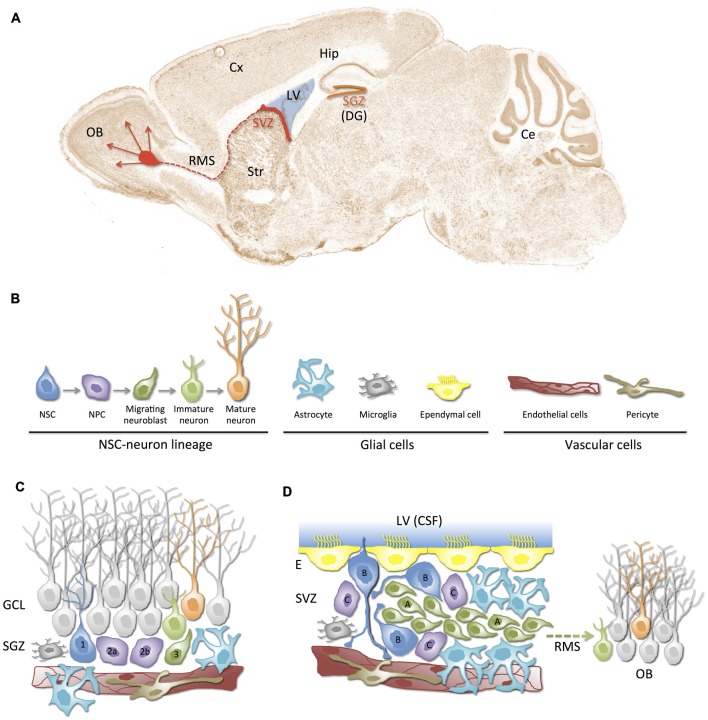
**Neurogenic niches in the adult mammalian brain. (A)** Schematic representation of the neurogenic regions (niches) in a sagittal section of the adult mouse brain: the subgranular zone (SGZ, orange) in dentate gyrus (DG) of the hippocampus (Hip), and the subventricular zone (SVZ, red) in the lateral wall of the lateral ventricles (LV). SVZ-derived newborn neurons migrate towards the olfactory bulb (OB) through the rostral migratory stream (RMS). **(B)** Cellular components of neurogenic niches. In addition to the neural stem cell (NSC)-neuron lineage, neurogenic niches are composed of glial cells (astrocytes, microglia and ependymal cells) and vascular cells (endothelial cells, pericytes). **(C,D)** Illustration of SGZ and SVZ neurogenic niches. The cytoarchitecture and relationships between cellular components of the niche are represented. Different cell types (color and shape) correspond to those depicted in **(B)**. Note the close proximity between blood vessels and NSCs/NPCs in both niches. **(C)** The SGZ neurogenic niche. Radial type 1 cells correspond to the NSCs that give rise to type 2a/b NPCs, which differentiate into type 3 neuroblasts. Neuroblasts migrate guided by astrocytes and become maturing neurons that finally mature and integrate into the granular cell layer (GCL). **(D)** SVZ neurogenic niche. This niche is located underneath the ependymal lining (E) of the LV. It is composed of type B quiescent cells (NSCs), which can activate and generate type C NPCs that rapidly proliferate and generate type A neuroblasts. Neuroblasts migrate long distances through the rostral migratory stream (RMS) to the OB where they mature into interneurons. Note that a tunnel of astrocytes and a scaffold of blood vessels guide migration of neuroblasts. Also note that monociliated type B cells can directly contact cerebrospinal fluid (CSF) and blood vessels. Ce, cerebelum; Cx, cortex; Str, striatum.

In the adult mammalian brain, neurogenesis is well documented to continue throughout life in two regions: the subventricular zone (SVZ) of the lateral ventricles (LV) and the subgranular zone (SGZ) of the dentate gyrus (DG) in the hippocampus (Figure 1A). A lineage model from NSCs to mature neurons has been characterized in both, the SVZ and the SGZ adult neurogenic niches (Figure 1B). In this model, NSCs give rise to neural progenitor cells (NPCs), also known as transit amplifying cells because of their limited division potential, which differentiate into migrating neuroblasts and then, into neurons that integrate into pre-existing circuits (Gage, 2000). Neuroblasts in the SGZ migrate short distances and integrate into the existing circuitry of the granular cell layer (GCL) of the DG (Figure 1C); those from the SVZ migrate along the rostral migratory stream (RMS) and supply newborn neurons for the olfactory bulb (OB; Figures 1A,D). Interestingly, glial and vascular cells are also major contributors to the configuration of functionally structured neurogenic niches (Figures 1B–D). Growing evidence suggests that the adult mammalian brain contains other neurogenic niches that are capable of generating new neurons and glial cells, particularly after injury or after some inductive stimuli (Lin and Iacovitti, 2015). The neocortex (Gould et al., 1999; Gould, 2007; Cameron and Dayer, 2008) and the hypothalamus (Kokoeva et al., 2005) have also been reported to support adult neurogenesis, but the magnitude of the neurogenic process in these regions is still under debate. Although the functional significance of adult-born neurons under physiological and/or pathological conditions has not been completely clarified and is being actively pursued, it is well-defined that neurogenesis in the SVZ and SGZ of the adult brain depends on the presence and maintenance of NSCs, which is tightly regulated by their highly specialized microenvironments or neurogenic niches (Palmer et al., 2000; Alvarez-Buylla and Lim, 2004; Ma et al., 2005; Merkle and Alvarez-Buylla, 2006).

Niches are defined by their ability to anatomically house stem cells and functionally control their development *in vivo* (Ma et al., 2008). The concept that stem cells reside within specific niches was first suggested in the 1970’s (Schofield, 1978), but it was not until the 2000’s, when substantial progress was made in describing both the cellular components of the niches and their functional interactions, in several mammalian tissues, including skin, intestine and bone marrow (Spradling et al., 2001; Li and Xie, 2005; Scadden, 2006). In the adult brain, much is known about the cellular composition and organization that characterize the SVZ and SGZ neurogenic niches (Ma et al., 2008; Mirzadeh et al., 2008; Aimone et al., 2014; Bjornsson et al., 2015; Licht and Keshet, 2015). Furthermore, the interaction and functional coordination of these components as well as the heterogeneity and complexity of neurogenic niches and their emerging roles under pathological conditions is being pictured (Jordan et al., 2007; Alvarez-Buylla et al., 2008).

### The Subventricular Zone (SVZ) Niche

Adult NSCs persist in a narrow niche along the walls of the LV, bordered on one side by the ependymal surface lining the cerebrospinal fluid (CSF)-filled ventricles and on the other by a complex arrangement of parallel blood vessels (Mirzadeh et al., 2008; Shen et al., 2008; Figure 1D). NSCs that reside in the SVZ, also known as Type B cells, exhibit hybrid characteristics of astrocytes (GFAP+) and immature progenitors (S100β+, Nestin+, Sox2+; Kriegstein and Alvarez-Buylla, 2009). Type B cell bodies are typically located under the ependymal lining of the LV and some of them have a short apical process with a single primary cilium that projects through the ependymal cell layer to contact the CSF directly, and a basal process that ends on the blood vessels of the SVZ plexus (Mirzadeh et al., 2008). Interestingly, apical processes of various type B cells form bundles at the center of a “pinwheel” of ependymal cells (Mirzadeh et al., 2008). As a result of their position and polarized phenotype, type B cells are strategically located to receive cues from both the vascular and the CSF compartments (Figure 1D). Quiescent type B cells can eventually divide asymmetrically to give rise to type C (Mash1+) transit-amplifying progenitor cells (Doetsch et al., 1997; Merkle and Alvarez-Buylla, 2006). Most of type C cells, in turn, divide to give rise to PSA-NCAM+ neuroblasts (type A cells). Type A cells form clusters and chains that migrate toward the OB guided by a channel of astrocytes and by a parallel scaffold of blood vessels. The anatomical structure formed by migrating (type A) neuroblasts is known as the RMS. Within the OB, these immature neurons differentiate into two types of GABAergic interneurons: the granular neurons and the periglomerular neurons, which integrate into the existing neuronal circuitry (Merkle and Alvarez-Buylla, 2006; Curtis et al., 2007; Kriegstein and Alvarez-Buylla, 2009; Figures 1A,D). Interestingly, type B/C cells can also originate glia (oligodendrocytes or astrocytes; Menn et al., 2006).

### The Subgranular Zone (SGZ) Niche

The SGZ is a region located beneath the GCL of the DG of the hippocampus (Figure 1C). The NSCs to mature neurons lineage model described in the SVZ can be comparably applied in the SGZ. A similar subset of GFAP+/Sox2+/Nestin+ radial glia-like cells are also believed to be quiescent NSCs of the SGZ (Seri et al., 2001). These NSCs, also known as type 1 cells, give rise through asymmetric division to transit-amplifying non-radial progenitors (Nestin+/GFAP−) or type 2a cells. Type 2a cells subsequently originate what appears to be a more fate-committed (Tbr2+) intermediate progenitor (type 2b) cell population, which give rise to doublecortin (Dcx) + (type 3) neuroblasts. Finally, neuroblasts differentiate into maturing glutamatergic granule cells that migrate, guided by astrocytic processes, and integrate in the GCL of the DG (Aimone et al., 2014; Figure 1C).

## Cellular Components of the Neurogenic Niche and their Role in the Neurogenic Process

**Figure 2:**
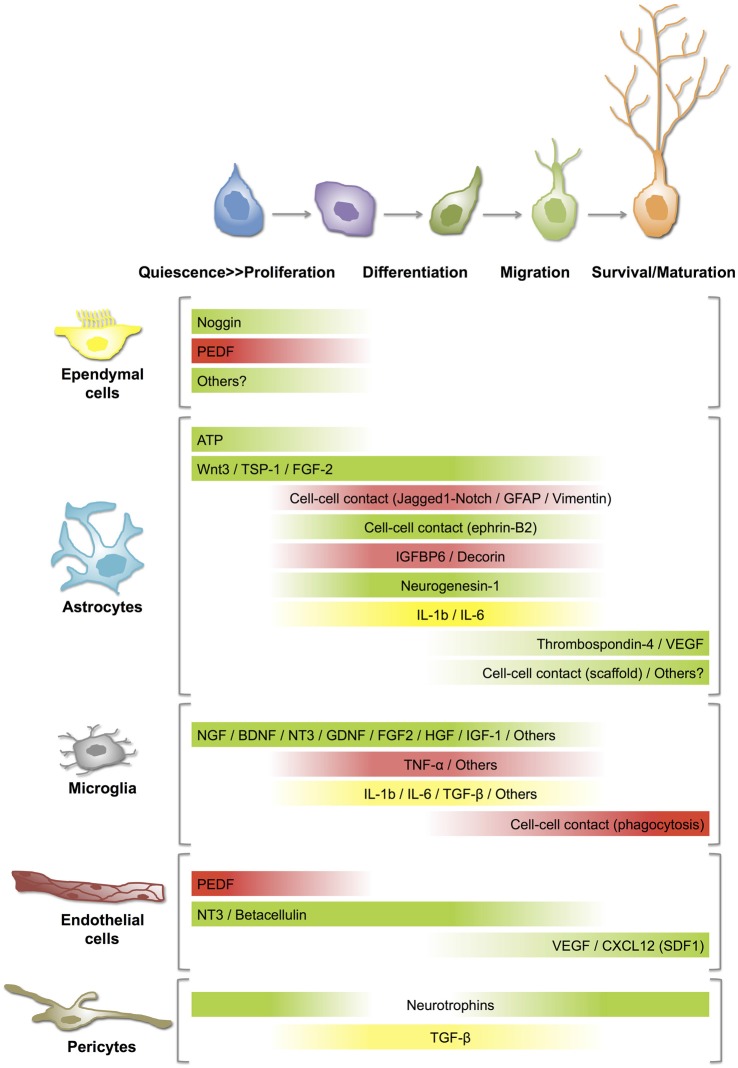
**Distinct roles of niche cells on NSC-neuron lineage at different stages/steps of adult neurogenesis.** Best-described cell-cell communication mechanisms in neurogenic niches rely on soluble mediators or direct contact between the signaling and the targeting cells. The gradient bars represent varying influence of secreted factors or cell-cell contacts on (i) activation of quiescent NSCs (proliferation); (ii) differentiation or fate specification; (iii) migration; and (iv) survival/maturation of newborn neurons. Green: stimulation or activation; Red: inhibition; Yellow: activation or inhibition according to the circumstances. For details and references, see the text. ATP, adenosine triphosphate; BDNF, brain-derived neurotrophic factor; CXCL, chemokine (C-X-C motif) ligand; FGF, fibroblast growth factor; GDNF, glial cell line-derived neurotrophic factor; GFAP, glial fibrillary acidic protein; HGF, hepatocyte growth factor; IGF, insulin-like growth factor; IGFBP, insulin-like growth factor binding protein; IL, interleukin; NGF, nerve growth factor; NT, neurotrophin; PEDF, pigment epithelium-derived factors; SDF, stromal cell-derived factor; TGF, transforming growth factor; TNF, tumor necrosis factor; TSP, thrombospondin; VEGF, vascular endothelial growth factor.

The highly hierarchical NSC-neuron lineage in both the SVZ and SGZ requires the precise regulation of NSCs self-renewal, fate specification, maturation and integration of new neurons in the existing neural circuitry. The convergence of several cellular and extracellular factors contributes to building a unique and specialized niche or microenvironment that regulates the physiology of NSCs during the course of adult life. Therefore, the identification and functional characterization of these factors emerges as a key aspect not only to better understand the biology of adult NSCs but also to develop novel therapies for a number of neurological and psychiatric disorders associated with defects in adult neurogenesis. Significant advances have been made in the description of the cellular components of the neurogenic niches and the mechanisms by which they can individually or coordinately contribute in regulating adult neurogenesis (reviewed in Ma et al., 2008; Aimone et al., 2014; Bjornsson et al., 2015; Figure 2).

### Astrocytes

Astrocytes represent one of the major contributors to the neurogenic niche (Song et al., 2002). Co-culture experiments with astrocytes isolated from the adult hippocampus and cortex, but not from the spinal cord, induce neuronal differentiation of NPCs (Barkho et al., 2006; Oh, 2010). Furthermore, hippocampus-derived astrocytes are more efficient than cortical astrocytes in promoting neuronal fate of NPCs (Oh, 2010), suggesting that the functional heterogeneity of astrocytes may be reflecting unique characteristics of different brain regions or environments. Interestingly, they may exert either positive or negative regulatory roles in the neurogenic process (Figure 2). Astrocytes negatively control neuronal differentiation of neural stem/progenitor cells through cell–cell contacts in which Jagged1-mediated Notch pathway and intermediate filament proteins GFAP and vimentin play a significant role (Wilhelmsson et al., 2012). Similarly, some astrocyte-secreted factors such as insulin-like growth factor binding protein 6 (IGFBP6) and decorin inhibit neuronal differentiation of adult NSCs/NPCs (Barkho et al., 2006). On the other hand, positive effects on neuronal differentiation of adult SGZ NSCs involve cell-cell contact by ephrin-B2(+) astrocytes (Ashton et al., 2012). Likewise, astrocytic ATP release positively regulates proliferation of NSCs in the SGZ (Cao et al., 2013) and astrocyte-derived soluble factors such as Wnt3 (Seri et al., 2001; Lie et al., 2005), neurogenesin-1 (Ueki et al., 2003), thrombospondin-1 (Lu and Kipnis, 2010) and interleukins such as IL-1β, IL-6 (Barkho et al., 2006), promote hippocampal neurogenesis. In agreement with a positive regulatory action of astrocytes, age-related changes in astrocyte population, including the decline of FGF-2 + astrocytes, correlate with low levels of neurogenesis in the aged hippocampus (Shetty et al., 2005).

Astrocytes also contribute to the migration of neuroblasts along the RMS by creating a physical route or tube where they (i) communicate with migrating neuroblasts (Bolteus and Bordey, 2004), and (ii) help to maintain the architecture of the vasculature scaffold in a VEGF- and thrombospondin-4-dependent fashion (Bozoyan et al., 2012; Girard et al., 2014). Similarly, in the adult SGZ, astrocytic radial processes facilitate the short-distance migration of newly generated neurons from the SGZ to the GCL of the DG (Shapiro et al., 2005). In addition, adult hippocampal astrocytes promote maturation and synaptic integration of the neural progeny of NSCs in co-culture experiments (Song et al., 2002).

### Ependymal Cells

Mature ependymal cells form a simple cuboidal to low columnar epithelium-like structure lining ventricular cavities. These cells have microvilli and tufts of motile cilia that contribute to the CSF hydrodynamic flow (Nelson and Wright, 1974; Ibañez-Tallon et al., 2004; Spassky et al., 2005). Disruption of ependymal lining or ciliary defects can provoke CSF flow disturbances and hydrocephalus (Jiménez et al., 2001; Wagner et al., 2003; Ibañez-Tallon et al., 2004; Banizs et al., 2005; Town et al., 2008). Because of their location, ependymal cells display certain barrier and signaling functions between CSF and neural tissue (Sarnat, 1992; Del Bigio, 1995). Ependymal cells are joined together at their apical regions by N-cadherin-based adherens junctions (Brightman and Reese, 1969; Del Bigio, 1995; Rodríguez and Bouchaud, 1996) and blocking N-cadherin function *in vitro* induces apoptosis of ependymal cells and denudation of ventricular walls (Oliver et al., 2013). Ependymal denudation of ventricular walls increases with aging (Luo and Craik, 2008) and is associated with the pathogenesis of neurodevelopmental disorders such as periventricular heterotopia (Ferland et al., 2009), spina bifida aperta (Sival et al., 2011) and hydrocephalus (Jiménez et al., 2009; Wagner et al., 2003; Domínguez-Pinos et al., 2005). Comprehensive analyses by Spassky et al. (2005) indicate that most ependymal cells are born at embryonic stages and that differentiated ependymal cells are postmitotic. However, it has been suggested that ependymal cells can be generated postnatally in certain brain regions (Bátiz et al., 2011). Furthermore, it has been argued that ependymal cells lining the SVZ can act as NSCs (Chojnacki et al., 2009). Interestingly, pathological stimuli such as stroke can stimulate forebrain ependymal cells to proliferate and generate neurons and astrocytes (Carlén et al., 2009).

Beyond this controversy, it is well defined that ependymal cells are a key component of the SVZ neurogenic niche. They are a source of noggin and pigment epithelium-derived factor (PEDF; Figure 2). Noggin is a bone morphogenetic protein (BMP) antagonist involved in the maintenance of a neurogenic niche (Lim et al., 2000). Opposite results have suggested that BMP signaling can inhibit adult neurogenesis (Lim et al., 2000), or promote it (Colak et al., 2008). Thus, the role of ependymal-derived noggin on the SVZ physiology is still not clear. PEDF is secreted by both ependymal and endothelial cells, and specifically participates in the self-renewal capacity of type B cells of the SVZ, thereby maintaining a pool of undifferentiated NSCs in the neurogenic niche (Ramírez-Castillejo et al., 2006). Furthermore, it is known that PEDF modulates stemness of NSCs by activating Notch-dependent transcription in these cells (Ramírez-Castillejo et al., 2006; Andreu-Agullo et al., 2009). In this context, the loss or disruption of the ependyma in the SVZ neurogenic niche would impair ependymal cell-mediated signaling pathways and alter its neurogenic potential. This appears to be the case in the SVZ niche of the hyh mutant mice where ependymal cells of the LV are completely lost during early postnatal stages and proliferation of SVZ progenitors is dramatically reduced (Jiménez et al., 2009). Interestingly, ependymal-denuded surfaces become progressively covered by a layer of astrocytes that acquire certain morphological and antigenic ependymal cell-like properties and probably contribute to the repair of the ependymal lining (Páez et al., 2007; Luo et al., 2008; Roales-Buján et al., 2012). However, the presence of a reactive astroglial “scar” can reduce the proliferative activity of SVZ neural progenitors, blocking neuronal regeneration and revascularization and thus, interfering with the recovery of an injured area (Fawcett and Asher, 1999; Kernie et al., 2001).

### Microglia

Most microglial cells of the CNS are already generated by the end of the second postnatal week (Ginhoux et al., 2013). In the adult brain, resting microglial cells represent the resident macrophages and they survey the brain parenchyma (Nimmerjahn et al., 2005). In the SGZ of the hippocampus, microglial cells have a prominent surveillant and phagocytic role. It has been demonstrated that most newborn granule neurons of the adult SGZ undergo apoptosis and are phagocytosed by microglial cells (Sierra et al., 2010, 2013). Beyond their role as brain’s professional phagocytes, microglial cells can also serve neuroprotective or neurotoxic roles depending on the physiological and pathological circumstances (Luo and Chen, 2012; Hellwig et al., 2013; Brites and Vaz, 2014). In addition, microglial cells can interact with NSCs/NPCs (Su et al., 2014) and regulate adult neurogenesis by secreting several soluble mediators, such as growth factors and cytokines, that influence either positively or negatively the neurogenic process (reviewed in Kim and de Vellis, 2005; Harry, 2013; Figure 2). *In vitro* experiments suggest that microglia secret factors that promote neuronal differentiation of SVZ-derived NSCs but not their maintenance or self-renewal (Walton et al., 2006). On the other hand, activation of microglial cells can result in negative regulatory actions on the SGZ neurogenic process (Sierra et al., 2014). For example, inflammatory or LPS-mediated activation of microglia can inhibit neurogenesis and favor gliogenesis and this effect is partially mediated by the secretion of proinflammatory cytokines, such as TNF-α (Monje et al., 2003; Butovsky et al., 2006; Carpentier and Palmer, 2009). On the other hand, when microglial cells are stimulated with interleukin-4 (IL-4) and interferon-γ, they secrete insulin-like growth factor-1 (IGF-1) and induce neuronal differentiation of neural progenitors (Butovsky et al., 2006). Additionally, the environmental context of the neurogenic niche determines not only the mode of activation of microglial cells and the positive or negative regulatory actions of these cells as a population but also defines the pro- or anti-neurogenic properties of specific microglial-secreted cytokines, such as the transforming growth factor β (TGF-β; Battista et al., 2006; Douglas-Akinwande et al., 2006). Interestingly, physical exercise (running)-induced cell proliferation and neurogenesis in the SGZ (van Praag et al., 1999b; Aimone et al., 2014) is associated to a decrease in microglial function (Olah et al., 2009; Vukovic et al., 2012; Gebara et al., 2013). Inversely, age-related changes in microglia activity, including an increase in their reactive profile with higher secretion of pro-inflammatory cytokines (Njie et al., 2012), potentially contribute to the decline of neurogenesis seen with aging (Gemma et al., 2010; Gebara et al., 2013).

### Endothelial Cells and Pericytes

The close physical proximity of NSCs/NPCs with blood vessels within both adult neurogenic niches suggests that critical factors derived from the vasculature may act as major modulators of the neurogenic process and consequently, several authors consider the SVZ and the SGZ as “vascular niches” (Palmer et al., 2000; Shen et al., 2008; Tavazoie et al., 2008; Licht and Keshet, 2015). Interestingly, vascular-derived signals or messengers can be originated distantly, in peripheral organs (blood-borne substances), and/or locally from endothelial cells and pericytes (paracrine communication). Endothelial cells are able to secrete several factors that modulate adult neurogenesis (Palmer et al., 2000; Shen et al., 2004; Figure 2). Several studies suggest that endothelial cell-produced brain derived neurotrophic factor (BDNF) and vascular endothelial growth factor (VEGF) stimulates SGZ neurogenesis both *in vivo* and *in vitro* (Jin et al., 2002; Cao et al., 2004; Kim et al., 2004). Interestingly, endothelial cells and NSCs reciprocally influence each other to couple SGZ neurogenesis to angiogenesis, and it is suggested that VEGF is critical to coordinate these processes (Palmer et al., 2000; Riquelme et al., 2008; Udo et al., 2008; Ruiz de Almodovar et al., 2009). Furthermore, VEGF appears to be necessary for exercise-induced SGZ neurogenesis (Fabel et al., 2003). Together with ependymal cells, endothelial cells secrete PEDF and thus, stimulate self-renewal of NSCs (Ramírez-Castillejo et al., 2006; Andreu-Agullo et al., 2009). Neurotrophin-3 (NT3) and betacellulin, secreted by vascular endothelial cells of both the SVZ plexus and the choroid plexus, regulate SVZ neurogenesis by different mechanisms. NT3 helps to maintain the quiescence of NSCs (Delgado et al., 2014), whereas betacellulin promotes proliferation of NPCs and neuroblasts (Gomez-Gaviro et al., 2012). Vascular endothelial cells also secrete a chemokine known as CXCL12 or stromal derived factor-1 (SDF1) that differentially modulates SVZ NSC-neuron lineage (Kokovay et al., 2010). In type B and type C cells, SDF1 upregulates EGFR and alpha-6 integrin, activating and attracting them to the blood vessels while SDF1 stimulates motility of type A neuroblasts (Kokovay et al., 2010).

The role of pericytes in adult neurogenic niches is less well characterized. Located in intimate contact with endothelial cells, they can act as regulators or transducers of both blood-circulating signals and endothelial-derived factors (Armulik et al., 2011). Pericytes secrete TGF-β, a known modulator of adult neurogenesis and blood-brain barrier (BBB; Dohgu et al., 2005). They also secrete neurotrophins in response to hypoxic conditions (Ishitsuka et al., 2012). In addition to their potential paracrine function, it has been demonstrated that pericytes remain relatively undifferentiated, retaining the capacity to differentiate into several cell types, including neural-related progeny. Consequently, it has been proposed that pericytes may act as NSCs under certain circumstances (Dore-Duffy et al., 2006; Dore-Duffy and Cleary, 2011). On the other hand, pericytes might be involved in the pathogenesis of different CNS diseases and have been proposed as a potential therapeutic target (Lange et al., 2013).

## Distant Regulation of the Neurogenic Niche: Neurotransmitters, CSF-Derived Factors, and Blood-Borne Substances

### Neurotransmitters (innervation)

Numerous neuromodulatory systems have been shown to affect proliferation and differentiation of NSCs/NPCs and the maturation of adult-born neurons. Adult SVZ receives inputs from dopaminergic projections from the ventral tegmental area (VTA) and the substantia nigra (Baker et al., 2004). It has also been shown that SVZ type C cells express D2-like dopaminergic receptors (Höglinger et al., 2004; Kippin et al., 2005). However, the effects of dopamine on adult NPCs are controversial and it has been suggested that dopamine differentially affects type B and C cells via a distinctive mix of receptors in each cell type (Berg et al., 2013). On the other hand, dopamine D2 receptor-induced neurogenesis appears to be mediated by ciliary neurotrophic factor (CNTF), a mitogen expressed by astrocytes that is upregulated by D2 agonists (Yang et al., 2008). The role of dopamine in the SGZ neurogenic process is less well understood (Veena et al., 2011) but it has been suggested that, similarly to the SVZ, D2 receptors are involved in hippocampal neurogenesis *in vivo* (Yang et al., 2008). Other major neuromodulatory systems include the serotoninergic and cholinergic systems. The role of serotonin in SGZ neurogenesis has been deeply studied because of its link to depressive-like behaviors (Gould, 2007). The DG receives serotoninergic inputs from the median and dorsal raphe nuclei (Leranth and Hajszan, 2007). Interestingly, it is well documented that fluoxetine, a serotonin-reuptake inhibitor widely used as antidepressant, increases the proliferation rates in the hippocampal SGZ (Malberg et al., 2000). Similarly, serotonergic axons originated in the raphe nuclei form a widespread network at the ventricular surface of the LV and contact type B NSCs of the adult mouse SVZ. Activation of serotonin receptors of Type B cells increases proliferation (Tong et al., 2014). Mouse SVZ NSCs/NPCs also received inputs from choline acetyltransferase-positive axons originating in the SVZ itself. The release of acetylcholine by these neurons depends on their activity and can directly control SVZ proliferation (Paez-Gonzalez et al., 2014).

In addition, local or regional network neurotransmitters also play a significant role in adult neurogenesis. It has been demonstrated that non-synaptic GABA signaling (released by type A neuroblasts) can regulate proliferation of type B NSCs in the SVZ by activating GABA_A_ receptors. This pathway provides a feedback mechanism to control the balance between self-renewal and differentiation of NSCs (Liu et al., 2005). Interestingly, this mechanism can also be reinforced by other SVZ GABAergic inputs such as the striatal GABAergic neurons (Young et al., 2014). In the SGZ niche, GABA helps to maintain the quiescence of radial glia-like (type 1) cells through activation of γ2-subunit containing GABA_A_ receptors (Song et al., 2012). Similarly to the SVZ, these GABAergic inputs appear to be non-synaptic and due to diffusion from nearby parvalbumin+ basket cells synapses (Song et al., 2012). On the other hand, type 2 NPCs and young neurons receive more direct GABAergic inputs (Ge et al., 2006; Markwardt et al., 2009). In the adult DG, maturing neurons also receive glutamatergic inputs and it has been shown that NMDA receptor activation is critical for the survival and integration of young neurons (Tashiro et al., 2006).

### CSF-Derived Factors

The SVZ is uniquely situated to experience the effects of the flow and the composition of the CSF. Intriguingly, the direction of migrating neuroblasts in the RMS, parallels that of CSF flow (Sawamoto et al., 2006). Furthermore, when ependymal ciliary movements and CSF flow are disrupted, neuroblasts become disoriented (Sawamoto et al., 2006). On the other hand, the CSF contains several compounds that participate in the neurogenic process (Lafon-Cazal et al., 2003; Bunn et al., 2005; Redzic et al., 2005; Zappaterra and Lehtinen, 2012). Transcriptome analyses of adult and embryonic choroid plexus (ChP) have revealed that ChP epithelial cells express several growth factors and signaling molecules that can act either as positive (TGF-α, amphiregulin, IGF2, and FGF2) or negative (TGF-β superfamily members) regulators of adult neurogenesis (Marques et al., 2011; Liddelow et al., 2012). They also secrete SLIT1/2, known chemorepulsive signals that help type A neuroblasts to migrate in the RMS (Nguyen-Ba-Charvet et al., 2004). Other factors, such as ciliary neurotrophic factor (CNTF) and leukemia inhibitory factor (LIF), play important roles in promoting proliferative activity and maintenance of undifferentiated neural progenitors (Shimazaki et al., 2001; Gregg and Weiss, 2005). Recently, it was found that two G-protein coupled receptor (GPCR) ligands present in the adult CSF, namely sphingosine-1-phosphate (S1P) and prostaglandin-D2 (PGD2), promotes quiescence of SVZ NSCs (Sato et al., 2007; Kondabolu et al., 2011; Codega et al., 2014). The role of the ChP/CSF-mediated signaling pathway has been highlighted in aged and hydrocephalic animal models. Aging-related changes in the ChP/CSF system negatively affect adult neurogenesis (Baruch et al., 2014). Additionally, it has been demonstrated that the composition of the CSF is altered in hydrocephalus (Mashayekhi et al., 2002; Owen-Lynch et al., 2003) and hydrocephalic CSF inhibits cell proliferation cortical cells cultures, suggesting that CSF composition modifies some of the properties of NPCs (Owen-Lynch et al., 2003). In fact, the altered composition of CSF affects the normal cortical development in hydrocephalic H-Tx embryos (Miyan et al., 2003). However, the specific CSF changes that mediate these effects are actually not known, and the influences of the hydrocephalic CSF on the SVZ neurogenic niche have not yet been reported.

### Blood-Borne Substances

As stated before, the vasculature can influence NSCs proliferation and differentiation by providing signaling molecules secreted locally by endothelial cells and pericytes, as well as by providing systemic blood-circulating factors (Tavazoie et al., 2008; Egeland et al., 2015; Licht and Keshet, 2015). Blood-borne factors that influence adult neurogenesis, including hormones, cytokines, metabolites, and gases can gain access to the SVZ neurogenic niche either via the ChP-CSF pathway route or more directly via the SVZ vasculature (Egeland et al., 2015; Licht and Keshet, 2015). In both cases, the presence of barriers must be considered. Indeed, ChP epithelial cells are joined together by tight junctions and constitute the blood-CSF barrier (BCB) while the presence of tight junctions between brain parenchymal endothelial cells constitute the BBB (Engelhardt and Sorokin, 2009). It has been suggested that SVZ vessels have a looser BBB than other brain vessels, allowing circulating substances to modulate adult neurogenesis. In this context, Tavazoie et al. (2008) have demonstrated that some circulating fluorescent tracers have better access to the brain parenchyma through SVZ vessels than through vessels located in other cerebral regions. Furthermore, NSCs/NPCs can directly contact blood vessels at specialized sites that lack pericyte coverage and glial end feet, a feature unique to SVZ vascular plexus (Tavazoie et al., 2008).

Several hormones seem to modulate adult neurogenesis under particular conditions. For example, prolactin can enhance SVZ neurogenesis in pregnant mice (Shingo et al., 2003), while increased levels of glucocorticoids associated with stress have the opposite effect (Snyder et al., 2011). In turn, dietary restriction and exercise/enriched environment positively modulate neurogenesis and it is proposed these effects are, at least in part, consequence of changes in the composition of blood-borne substances (van Praag et al., 1999a; Wu et al., 2008). Interestingly, the age-related decline of the neurogenic niche could be restored by extrinsic young signals. Using a mouse heterochronic parabiosis model, Katsimpardi et al. (2014) showed that (i) aged cerebral vasculature is remodeled, and (ii) SVZ neurogenesis is re-activated in response to young systemic factors. Furthermore, the authors revealed that GDF11, a circulating TGF-β family member, participate in this process (Katsimpardi et al., 2014). These examples emphasize the fact that the adult neurogenic niche communicates with the systemic circulation and highlight the role of blood-borne substances in the modulation of adult neurogenesis.

Interestingly, in addition to the well-recognized soluble signals originated both locally by the cellular components of the adult neurogenic niches (reviewed in “Cellular components of the neurogenic niche and their role in the neurogenic process” Section), or distantly (reviewed in “Distant regulation of the neurogenic niche: neurotransmitters, cerebrospinal fluid-derived factors, and blood-borne substances” Section), novel types of intercellular messengers, such as EVs, have emerged. EVs, and particularly exosomes, are considered one of the most complex ways of cell-to-cell communication, allowing the transfer of mRNAs, microRNAs (miRNAs), proteins and lipids between cells and thus, being able to modify the physiology of recipient cells. However, their precise role in adult neurogenic niches remains virtually unexplored.

## Exosomes: Unique Messengers for Cell-to-Cell Communication

EVs, and particularly exosomes, are emerging as one of the major mediators of intercellular communication. It is known since the late 1960’s/early 1970’s that membrane-enclosed vesicles are present outside cells in different tissues and biological fluids (Colombo et al., 2014). Originally, these EVs were thought to be released only by outward budding or shedding of the plasma membrane (PM) but 10 years later, it was described that vesicles contained within the lumen of so-called multivesicular endosomes or multivesicular bodies (MVBs) could be secreted (Harding and Stahl, 1983; Pan and Johnstone, 1983). Thus, the term “exosome” was introduced to identify those EVs that have been originated from the endosomal system (Johnstone et al., 1987) and since then, the field of exosome research has expanded exponentially. Now it is known that various types of EVs coexist in the extracellular milieu. Some of them are originated from shedding of the PM (microvesicles and apoptotic bodies) and others (exosomes) are secreted by exocytosis after fusion of MVBs with the PM. Thus, the various intracellular origins and modes of formation can probably lead to different compositions and functions of EVs (Kowal et al., 2014). Exosomes represent one sub-type of these EVs and a lot of progress has been made in the last few years to understand the basic mechanisms by which exosomes are formed and secreted, and their function in cell-cell communication under physiological and pathological conditions.

### Biogenesis of Exosomes

**Figure 3:**
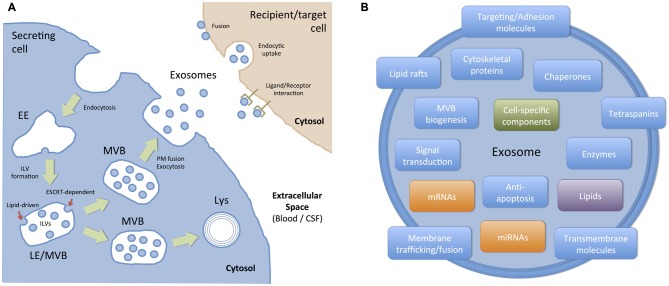
**Biogenesis, targeting and composition of exosomes. (A)** Exosomes are presumed to be a relatively homogeneous population of vesicles originated in the endocytic pathway as intraluminal vesicles (ILVs) of late endosomes (LE) or multivesicular bodies (MVBs). Basically, ILVs are formed by the inward budding of the membrane of maturing early endosomes (EE) to MVBs. Cargo sorting into ILVs include: (i) ESCRT-dependent mechanisms, where the endosomal sorting complex required for transport (ESCRT) and other associated proteins are involved, and (ii) ESCRT-independent (lipid-driven) mechanisms, which depends on the presence of ceramide and neutral sphingomyelinase. Then, a population of MVBs is destined to degradation into lysosomes (Lys) and another population of MVBs fuse with the plasma membrane (PM), thus allowing ILVs to be exocytosed as exosomes. Once in the extracellular space, secreted exosomes can act as local signals (paracrine communication) or travel through biological fluids (e.g., blood, CSF) to reach body sites located distant from their cell/tissue of origin. Exosomes interact with recipient/target cells in several ways: (i) they can activate surface receptors (ligand/receptor interaction); (ii) they can transfer exosomal cargo to recipient cells by membrane fusion or by a connexin-dependent mechanism; and (iii) they can be endocytosed by a macropinocytic mechanism and then fuse with endosomal membranes to transfer their cargo. **(B)** Exosomes are small (30–100 nm) membrane bound vesicles with a complex and functionally relevant composition. They contain nucleic acids (mRNA and miRNA), and a vast array of different proteins and lipids depending on their host cell. However, they are generally enriched in proteins involved in MVB formation, tetraspanins, membrane trafficking and fusion, and a number of cytosolic proteins. In addition to these generic components, molecules associated with particular cell types and/or pathological situations have also been identified in exosomes.

Exosomes are small membrane vesicles, 30–100 nm in size, that are secreted by almost all cell types. They originate in the endocytic pathway, which is involved in the trafficking of several proteins that are internalized and can either recycle back to the PM or get sorted to degradation (Gould and Lippincott-Schwartz, 2009; Klumperman and Raposo, 2014) In these pathways, early endosomes mature into late endosomes (LE), and during this process, they suffer an inward budding process of their membrane and accumulate intraluminal vesicles (ILVs; Figure 3A). Because of their mature morphological features, LEs are referred to as MVBs. In most cells, the main fate of MVBs is to fuse with lysosomes, ensuring the degradation of their content. However, organelles with hallmarks of MVBs, can also fuse directly with the PM, releasing ILVs into the extracellular milieu (Raposo et al., 1996; Jaiswal et al., 2002; Figure 3A). To date is still unknown how different subpopulations of MVBs co-exist within cells and whether these different populations follow the same or different routes such as fusion with lysosomes for degradation or fusion with PM for exocytosis (Edgar et al., 2014). The best-described mechanism for the formation of MVBs and ILVs is driven by the endosomal sorting complexes required for transport (ESCRT) machinery. This machinery is composed of approximately thirty proteins that assemble into four complexes (ESCRT-0, -I, -II and -III) with associated proteins (VPS4, VTA1, ALIX also called PDCD6IP) conserved from yeast to mammals (Hanson and Cashikar, 2012). The ESCRT-0 complex recognizes and sequesters ubiquitinated transmembrane proteins in the endosomal membrane, segregates them into microdomains and binds the ESCRTI complex. ESCRTI, in turn, recruits ESCRTII subunits, and then both complexes initiate membrane deformation into buds with sorted cargo, allowing cytosolic proteins and RNAs (including mRNAs and miRNAs) to get into the forming vesicles. Following, the ESCRTII complex recruits ESCRTIII subunits inside nascent vesicles and together with the ATPase VPS4, provoke the membrane cleavage required to free ILVs while ubiquitin molecules and ESCRT subunits are released into the cytosol for their recycling (Hanson and Cashikar, 2012). Recently, alternative ESCRT-independent mechanisms have been proposed for the formation and sorting of specific cargo into exosomes, including a lipid-driven mechanism in which the synthesis of ceramide by a neutral sphingomyelinase is involved (Trajkovic et al., 2008) and a sphingosine 1-phosphate receptor-dependent mechanism (Kajimoto et al., 2013). Alternatively, ALIX, an ESCRTIII binding protein, by interacting with proteins containing late-domain motif LYPXnL, such as syntenin or the GPCR Par1, can be sorted into exosomes by a mechanism that requires only the recruitment of the ESCRTIII subunit CHMP4 (Sette et al., 2010; Baietti et al., 2012; Hurley and Odorizzi, 2012).

### Content and Extracellular Fate of Exosomes

Considering the mechanisms involved in the biogenesis of ILVs, a complex mix of proteins, lipids and other cytosolic molecules is specifically sorted and incorporated into ILVs/exosomes.The content of exosomes is currently compiled in databases named Exocarta^1^, Vesiclepedia^2^ and EVpedia^3^ which are continuously updated by the scientific community (Mathivanan et al., 2012). According to the current version of Exocarta, 9769 proteins, 1116 lipids, 3408 mRNAs, and 2838 miRNAs have been identified in exosomes from many different cell types and organisms. Much effort has been made to establish the proteomics of exosomes. Initially, it was found that exosomes contain proteins also present in endosomes, the PM, and the cytosol, but few components from other organelles such as nucleus, mitochondria or Golgi, confirming that they are not composed of a random set of proteins (Théry et al., 2001). The most frequently identified proteins in exosomes includes (i) proteins involved in MVB biogenesis; (ii) membrane associated proteins (lipid rafts proteins, GTPases and other membrane trafficking proteins); (iii) transmembrane proteins (targeting/adhesion molecules, tetraspanins (CD9, CD63, CD81), membrane fusion proteins); (iv) cytoskeletal proteins (actin, syntenin, moesin); (v) signal transduction proteins (annexin, 14–3–3 proteins); (vi) chaperones (Hspa8, Hsp90); and (vii) metabolic enzymes (GAPDH, LDHA, PGK1, aldolase, PKM), among others (Chaput and Théry, 2011; Mathivanan et al., 2012; Figure 3B). Interestingly, and even though there is an important overlap in terms of protein composition with other EVs, many of these proteins, such as tetraspanins, CD63 and CD81, are considered exosome markers.

In addition, exosomes contain a specific lipid composition (Kowal et al., 2014): they are enriched in ceramide, cholesterol, sphingomyelin, and phosphatidyl-serine. Interestingly, they are not enriched in lysobisphosphatidic acid, a lipid described in ILVs (Matsuo et al., 2004). As stated before, some of these lipids can be involved in the formation of ILVs or their release outside the cell. One very striking aspect of the content of exosomes is that they are highly enriched in small (no longer than 200 nucleotides) non-coding RNAs (ncRNAs). The most widely described exosome-associated ncRNAs are the miRNAs of 20–22 nucleotides that target the 3′ untranslated region (UTR) of specific mRNAs to inhibit, in most cases, their translation. As a consequence of this, miRNAs can modify the phenotype and/or the physiology of the recipient cell, modulating cellular processes as relevant as proliferation, differentiation, and survival, among others (Cocucci and Meldolesi, 2015). The mechanisms that control the incorporation of the cargo into exosomes are currently under intense investigation (Villarroya-Beltri et al., 2014). Importantly, besides a “constitutive” array of proteins, lipids and RNAs, the content of exosomes varies according to the cellular source and to the physiological or pathological situation of the cell and its environment. Thereby, exosomes serve as interpreters of the cellular state where they came from. The role of exosomes as intercellular messengers has been studied extensively using cells of the immune system (Bobrie et al., 2011), where it has been demonstrated that they modulate antigen presentation and the immune response (Théry et al., 2009). Similarly, the role of exosomes in several cancer types has been well documented. For instance, glioblastoma-derived exosomes deliver genetic information to recipient endothelial cells, promoting tumor growth and invasion (Skog et al., 2008).

Once secreted, exosomes can interact with target cells to modify their function (Mittelbrunn and Sánchez-Madrid, 2012) and thus, they can act as local or paracrine messengers or communicators. Also, they can reach biological fluids, such as blood, CSF, urine, etc., and act as distant messengers (Figure 3A). The targeting of exosomes to their recipient cells involves different mechanisms (Colombo et al., 2014; Mulcahy et al., 2014). In some cases, ligand/receptor interaction of EVs to target cells might be sufficient to activate intracellular pathways and induce physiological changes in target cells. In other cases, for instance when miRNAs contained in exosomes induce gene silencing in the recipient cell, the content of EVs must be transferred inside that cell. In those cases, fusion of exosomes with the PM or endocytosis followed by fusion of exosomes with the membrane of endocytic compartments must take place (Figure 3A). Microenvironmental pH appears to be relevant for the fusion of exosomes with endosomal membranes (Parolini et al., 2009). Alternatively, a novel connexin 43-mediated mechanism to transfer information between exosomes and recipient cells has recently been described (Soares et al., 2015). According to this paper, the content of exosomes can be transferred to recipient cells by a gap junction-like communication, thus opening new possibilities about the mechanisms that might operate in specific environments or situations.

## Can Exosomes Serve as (Physiological or Pathological) Messengers Between Cellular Components of the Adult Neurogenic Niche?

An efficient and well-regulated communication between cells is vital to ensure brain homeostasis and plasticity throughout life, particularly in the adult neurogenic niches. Thus, information transfer through exosomes (and other EVs) appears as a unique mechanism compared with other forms of intercellular communication. Even though the physiological and/or pathological role of locally- or distantly-generated exosomes in the adult neurogenic niches is currently virtually unknown, growing indirect evidence strongly suggest that exosomes might be one the major elements communicating and coordinating the function of the adult neurogenic niches. First, neural stem/progenitor cells and most of the cell types present in the CNS, including those cells that constitute and regulate the neurogenic niche, secrete and/or are target of exosomes and other EVs (Table 1). Furthermore, some of the biomolecules expressed (and sercreted) by niche cells have been reported to be present in exosomes under physiological or pathological conditions (Table 1). Interestingly, several examples highlight the role of exosomes as (i) messengers between neural cells (neurons and glial cells) either locally or distantly (via CSF or volume transmission); (ii) blood-CNS communicators (including their potential as therapeutic vehicles); and (iii) modulators of several stem cell niches.

**Table 1:** **Niche cells (and other CNS cells) secrete exosomes with potential physiological and pathological functions**.

Niche cells	Examples of biomolecules expressed by niche cells and found in exosomes (*)	Physiological function	Pathological function	Reference
NSCs/NPCs	T-cell immunoglobulin mucin protein 4 (TIM-4) Interferon gamma (IFN-*γ*)/IFN-*γ* receptor 1 (Ifngr1) Heat shock protein 70 KDa (Hsp70) VCAM1 **Connexin 43 (Cx43)** **VEGF** **miR-let7b** **miR-9**	Regulate NSC proliferation and differentiation (miR-let7b and miR-9) (Zhao et al., 2009, 2010) Favor exosome targeting and transfer of information to acceptor cells (Cx43) (Soares et al., 2015) Regulate the SGZ neurogenic niche (VEGF) (Kirby et al., 2015) Maintain protein homeostasis and regulates cell survival (Hsp70) (Takeuchi et al., 2015) Modulate gene expression in target cells via Stat1 activation (IFN-*γ*) (Cossetti et al., 2014)	Mediate viral entry (TIM-4) (Sims et al., 2014) Modulates immune response (Cossetti et al., 2014) Autoimmunity (Kang et al., 2008) Neurophatological development of NSCs/NPCs (Feliciano et al., 2014)	Marzesco et al. (2005) Huttner et al. (2008) Kang et al. (2008) Kunze et al. (2009) Akerblom and Jakobsson (2013) Bian et al. (2013) Drago et al. (2013)
Neurons	p75 (neurotrophin receptor) Nedd4 family proteins and Nedd4 family-interacting protein 1 (Ndfip1) miR-124a Cystantin C L1 cell adhesion molecule (L1-CAM) GPI-anchored prion protein GluR2/3 (glutamate receptors subunits) Hsp70 α-synuclein PrPsc APP **miR-34a**	Regulate neuronal physiology: neurite outgrowth, cell death/survival balance (p75). Escudero et al. (2014) Neuroprotection (e.g., removal of proteins during stress; Nedd4) (Putz et al., 2008; Low et al., 2015) Regulate translation of glutamate transporter GLT1 in astrocytes (Morel et al., 2013) and neuronal fate in SVZ NSCs (miR-124a) Cheng et al. (2009) and Akerblom et al. (2012) Regulate proliferation and differentiation of adult NSCs (Cystatin C) (Ghidoni et al., 2011) Regulatory function at synapses (Faure et al., 2006) Stimulate microglial phagocytosis (synaptic pruning) (Bahrini et al., 2015) Regulate NSCs differentiation, neuroblasts migration and neuron maturation (miR-34a) (Mollinari et al., 2015) Volume transmission (Agnati and Fuxe, 2014)	Vehicles for the transfer of toxic proteins (PrPsc: Prion disease; APP: Alzheimer’s disease; superoxide dismutase: amyotrophic lateral sclerosis (ALS); alpha-synuclein: Parkinson’s disease) Fevrier et al. (2004); Alvarez-Erviti et al. (2011b) and Bellingham et al. (2012b) miRNAs can stimulate inflammatory response (activating Toll-like receptors) in stroke, ALS and other neurodegenerative diseases (Paschon et al., 2015) miR-124 dysregulation is associated to several CNS disorders Sun et al. (2015) Cystatin C dysregulation is associated to Alzheimer’s disease Ghidoni et al. (2011)	Smalheiser (2007) Lachenal et al. (2011) Von Bartheld and Altick (2011) Smythies and Edelstein (2013) Chivet et al. (2012, 2013, 2014) Ryan et al. (2015)
Astrocytes	Prostate Apoptosis Response 4 (PAR-4) Ceramide Synapsin I FGF-2 VEGF PEDF Hsp70 **IGFBP6** **miR-125b** **Cx43**	Promote neurite outgrowth and neuronal survival (synapsin I) (Wang et al., 2011) Induce glial cell proliferation (miR-125b) (Pogue et al., 2010) Favor exosome targeting and transfer of information to acceptor cells (Cx43) (Soares et al., 2015) Angiogenesis. Proliferation and differentiation of varios cell types (FGF-2, VEGF) (Proia et al., 2008) Regulate stemness of NSCs (PEDF) (Ramírez-Castillejo et al., 2006) Volume transmission(Agnati and Fuxe, 2014)	Amyloid-induced exosomes are enriched in ceramide and PAR-4. Apoptosis induction in Alzheimer’s disease (Wang et al., 2012) Astrogliosis. Alzheimer’s disease, Down’s syndrome (miR-125b) (Pogue et al., 2010)	Taylor et al. (2007) Guescini et al. (2010) Frühbeis et al. (2012) Hajrasouliha et al. (2013) Agnati and Fuxe (2014)
Oligodendrocytes (**)	Superoxide dismutase, catalase Myelin proteins (CNP, MBP, MOG, PLP) Lipids (galactocerebroside, sulfatide, cholesterol)	Enhance neuronal stress tolerance, promote neuronal survival during oxygen/glucose deprivation, and regulate neuronal physiology (increase firing rate, modulate gene expression and signal transduction pathways) (Frühbeis et al., 2013a; Fröhlich et al., 2014) Trophic support for axons (Krämer-Albers et al., 2007) Oligodendrocyte-microglia communication (Fitzner et al., 2011)	Neuroinflammation (Gupta and Pulliam, 2014) Autoantigen in multiple sclerosis (PLP) (Krämer-Albers et al., 2007)	Hsu et al. (2010) Lopez-Verrilli and Court (2013) Peferoen et al. (2014)
Microglia	Surface-bound aminopeptidase N (CD13) Monocarboxylate (lactate) transporter 1 (MCT1) Metabolic enzymes (Gliceraldehyde- 3-phosphate dehydrogenase) **miR-155** **Growth Factors (FGF2, IGF1, BDNF)**	Neuropeptide (enkephalins) catabolism (CD13) (Potolicchio et al., 2005) Supportive, neuroprotective role (Hooper et al., 2012; Prada et al., 2013) Exosome release is modulated by serotonin and Wnt3a (Hooper et al., 2012; Glebov et al., 2015) Modulate proliferation and differentiation of NSCs /NPCs (growth factors) (Grote and Hannan, 2007; Ma et al., 2009)	Propagation of inflammation signals. Neurodegenerative diseases (Prada et al., 2013) Inflammation-induced hippocampal neurogenic deficits (Woodbury et al., 2015)	Bianco et al. (2005) Potolicchio et al. (2005) Bianco et al. (2009) Tamboli et al. (2010) Turola et al. (2012) Su et al. (2014) Gomez-Nicola and Perry (2015)
Endothelial Cells	Delta-like 4 (Dll4), (membrane-bound Notch ligand). miR-126 miR-214 miR-296 (angiomirs) **Growth Factors (VEGF, PEDF)**	Induce angiogenesis (miR-126, miR-214 and Dll4) (Sheldon et al., 2010; van Balkom et al., 2013; Sharghi-Namini et al., 2014; van Balkom et al., 2015) Increase levels of pro-angiogenic receptors (miR-296) (Würdinger et al., 2008) Modulate proliferation and differentiation of NSCs/NPCs (growth factors) (Grote and Hannan, 2007)	Cellular stress changes RNA and protein composition of endothelial cell-derived exosomes. Transfer of stress signals (hypoxia, inflammation, hyperglycemia) to target cells (de Jong et al., 2012) Inflammation-induced EVs (exosomes?) induce changes in protein expression pattern of cerebrovascular pericytes (Yamamoto et al., 2015)	Shen et al. (2004) Simak et al. (2006) Tavazoie et al. (2008) Jung et al. (2009) Haqqani et al. (2013) Crouch et al. (2015) van Balkom et al. (2015)

(*) In bold are presented those biomolecules expressed by the referred cell type and found in exosomes of other cell types. (**) Oligodendrocytes are not typically considered niche cells but are included here as another example of CNS cell-to-cell communication via exosomes.

### Exosomes in Neuron-Neuron Communication

Classical inter-neuronal communication involves synaptic transmission, a dynamic and plastic process that is tightly regulated by neuronal activity (Regehr et al., 2009). Exosome-mediated communication between pre and postsynaptic cells participates in synaptic plasticity, as it has been shown in the *Drosophila* neuromuscular junction (Korkut et al., 2013). Using cultures of mixed hippocampal cells with exosomes derived from the neuroblastoma cell line N2a and labeled with GFP-CD63 and GFP-TTC, it was found that they interact either with neurons, astrocytes or oligodendrocytes. On the other hand, exosomes released by cortical neurons upon synaptic activation interact with neurons but not with GFAP+ astrocytes. Furthermore, some exosomes co-localize with synaptophysin indicating that they bind to pre-synaptic sites (Chivet et al., 2014).

### Exosomes in Neuron-Glia Communication

The communication between neurons and glia is important for brain physiology during both development and adulthood. The different glial cell types help to maintain neuronal activity. Oligodendrocytes protect axons with the myelin sheath and also provide trophic support to neurons (Nave and Trapp, 2008). To maintain these functions over time there is a constant communication between neurons and oligodendrocytes, but the mechanisms underlying this phenomenon are not well understood. Frühbeis et al. demonstrated that upon glutamate stimulation, oligodendrocytes secrete exosomes, which are endocytosed by neurons. Furthermore, exosomal cargoes improve neuronal metabolism and viability in situations of nutrient deprivation or oxidative stress exposure (Frühbeis et al., 2013b). It is also noteworthy that this work demonstrated that the internalization of exosomes by neurons occurs through a clathrin and dynamin-dependent mechanism, shedding light on the mechanisms that may be involved in exosome internalization. On the other hand, selective elimination of synaptic connections comprises the engulfment of neurites. In a recent study, it was shown that neuron-derived exosomes stimulate microglial phagocytosis of neurites via upregulation of complement factors (Bahrini et al., 2015).

### Exosomes in Glia-Glia Communication

The communication between glial cells through exosomes has been studied to a lesser extent. Exosomes secreted by oligodendrocytes are selectively internalized through macropinocytosis by microglia, both *in vitro* and *in vivo* (Fitzner et al., 2011). Remarkably, only those microglial cells that do not show antigen-presenting capacity endocytose exosomes, thus supporting the idea that different types of microglial cells co-exist and are differentially involved in immune functions.

### Exosomes in the CSF as Volume Transmission Vehicles

It has been proposed that the CSF compartment plays an essential role in volume transmission within the CNS; thus, molecules or messengers secreted in one brain region may reach the CSF and exert their function in sites located far from its secretion site (Agnati and Fuxe, 2014; Borroto-Escuela et al., 2015). Given the close contact between the CSF and the interstitial fluid of several brain areas, including the SVZ, it is conceivable that exosomes originated in the brain parenchyma can be found in the CSF and *vice versa*. Actually, isolation of membrane vesicle-enriched fractions and further proteomic studies have demonstrated the presence of exosomes in the human CSF (Street et al., 2012; Grapp et al., 2013; Chiasserini et al., 2014). Furthermore, the exosome content of the CSF is supposed to reflect ongoing brain processes, and especially those related to plasticity, disease or repair. Proteins related to the onset or progression of some CNS diseases such as APP (Alzheimer’s disease), PrPsc (prion disease), and α-synuclein (Parkinson’s disease), among others, have been found in the exosomal fraction of CSF-samples (Pegtel et al., 2014). Exosomes in the CSF decrease with age while those derived from the embryonic CSF positively act on the stem cell niche (Street et al., 2012; Feliciano et al., 2014), revealing their influence on recipient cells. A clear demonstration of exosomal secretion into the CSF has been recently obtained in ChP epithelial cells. Using cell-culture assays, human CSF analyses and *in vivo* tracing experiments, the authors describe a novel pathway of exosome-mediated folate delivery into the CSF and subsequently, into the brain parenchyma (Grapp et al., 2013).

### Exosomes in the Blood to CNS Communication and as Therapeutic Vehicles

Exosomes constitute one of the most attractive vehicles to communicate peripheral organs with the CNS and *vice versa*. The fact that blood-circulating exosomes may reach and be incorporated into different organs has stimulated scientists from diverse disciplines to explore the use of exosomes as therapeutic vehicles able to deliver specific drugs (Suntres et al., 2013). This widespread interest includes the use of exosomes as therapeutic agents in cancer (Pitt et al., 2014; Greening et al., 2015; Tran et al., 2015), and in infectious and allergic diseases (Admyre et al., 2008; Prado et al., 2008; Hosseini et al., 2013), to give just a few examples. Exosomes are considered attractive vehicles of blood to CNS communication due to (i) their stability (their cargo is protected from RNAses and proteases); (ii) their lack of immunogenicity (when derived from the same patient); (iii) the possibility of adding surface proteins or antibodies to target specific cell types; (iv) the possibility of loading a specific molecular cargo with therapeutic actions; and (v) importantly, their capacity to cross the BBB (Aryani and Denecke, 2014; Ridder et al., 2014; György et al., 2015; Kawikova and Askenase, 2015). siRNAs, for example, have been successfully targeted to specific brain regions in mice following systemic injection of siRNA-electroporated exosomes (Alvarez-Erviti et al., 2011c; El-Andaloussi et al., 2012). This strategy has been reported to be effective as a way to decrease α-synuclein aggregates in wild type as well as in transgenic mice expressing the phospho-mimic S129D α-synuclein, which is prone to aggregation (Cooper et al., 2014). Exosomes have also been used to deliver curcumin and JSI124 (activator of transcription 3 inhibitor) to brain microglia of mice via an intranasal route, protecting from inflammation and delaying tumor growth (Zhuang et al., 2011). These examples emphasize the potential of exosomes, not only as therapeutic vehicles, but also as physiological and pathological messengers between peripheral organs and the CNS. Even though the mechanisms by which peripheral exosomes have access to the brain tissue are unknown, the fact that adult NSCs reside in vascular niches, leads to the interesting proposal that blood-borne exosomes may influence adult neurogenic niches.

### Exosomes as Modulators of Diverse Stem Cell Niches

Cancer stem cells (CSCs) or cancer initiating cells (CICs) are tumor cells that have properties of self-renewal, clonal tumor initiation, and metastatic potential (Zhang et al., 2015b). As other stem cells, CSCs reside in distinct regions within the tumor called niches, which protect CSCs from immune responses and preserve their phenotypic plasticity (Plaks et al., 2015). Furthermore, the niche is believed to play a pivotal role in the resistance of CSCs to some cancer therapies (Kuhlmann et al., 2015; Plaks et al., 2015). It is known that exosomes released locally from tumor cells are able to modify the niche, promoting angiogenesis and tumor cell proliferation (Tickner et al., 2014). In addition, cancer-derived exosomes can travel to sites located outside the tumor to induce cellular changes associated with the promotion of a “pre-metastatic” niche, a special microenvironment that is able to receive and harbor cancer cells, thus favoring metastasis. This has been demonstrated in the case of melanoma metastasis in the bone marrow (Peinado et al., 2012) and liver metastasis of pancreatic ductal adenocarcinoma (Costa-Silva et al., 2015). Even though the mechanisms by which exosomes modulate primary tumor and pre-metastatic niches are not fully understood, it has been shown that isolated exosomes from body fluids of cancer patients (blood, ascites fluid and urine, CSF) contain several growth factors and cytokines able to modulate the environment of the metastatic niche (Ung et al., 2014).

On the other hand, non-tumoral mesenchymal stem cells (MSCs) are extensively used in different cell therapy-based clinical trials today. It is known that functional improvement with MSCs therapies is not mainly due to cell engraftment or differentiation at the site of injury but they exert their effects through their secreted products, including exosomes (Lai et al., 2015). Exosomes harvested from the conditioned media of MSCs cultures increase angiogenesis and neurogenesis, and promote functional recovery in animal models of stroke and traumatic brain injury (Xin et al., 2013a,b; Zhang et al., 2015c). Thus, in therapies for brain disorders, it has been proposed that exosomes derived from MSCs function as an extension of MSCs, and like their cell-source, exosomes can target biological processes that stimulate functional repair of the damaged nervous system (Lai et al., 2015). However, it is thought that similar exosomes isolated from MSCs of different origins (i.e., bone marrow, menstrual, chorion or umbilical cord) might differentially affect target cells, possibly due to a differential molecular cargo (Lopez-Verrilli and Court, 2013). In this context, albeit a direct participation of exosomes in the adult neurogenic niches has not yet been addressed, it is conceivable that exosomes derived from the different cell types of the neurogenic niche as well as the heterogeneous exosome mixture that may reach the niche via CSF-volume transmission or through the vasculature, affect the neurogenic process in a differential and highly specific manner. Given the relevance of these actions to a large array of diseases of the nervous system, the cellular and molecular mechanisms involved in the regulatory functions of exosomes in the neurogenic niche are an attractive field for future investigations.

## Molecular Components of Exosomes with a Potential Role in the Regulation of the Neurogenic Niche

### MicroRNAs

Several steps of adult neurogenesis are mediated or regulated by miRNAs. For example, miR-let7b and miR-9 regulate proliferation and differentiation of NSCs (Zhao et al., 2009, 2010). Similarly, miR-34a regulates NSCs differentiation, neuroblast migration and neuron maturation (Mollinari et al., 2015). miR-124a is a key determinant of neuronal fate of SVZ NSCs by targeting Sox9 (Cheng et al., 2009; Akerblom et al., 2012) or by targeting the JAG-Notch signaling pathway (Liu et al., 2011). On the other hand, miR-128 overexpression reduces the levels of doublecortin (Dcx) in differentiating NPCs, indicating that miR-128 can target and potentially take part in the regulation of Dcx levels in adult neurogenesis (Cernilogar et al., 2015). Furthermore, it has been recently shown that miR-124, miR-128 and miR-137, can act cooperatively and synergistically to promote neuronal differentiation of NSCs by targeting overlapping gene sets containing a highly interconnected transcription factor network (Santos et al., 2015). miRNAs are also involved in glial cell proliferation/differentiation and angiogenesis, phenomena closely related and associated with adult neurogenesis. For example, miR-125b is involved in glial cell proliferation under physiological and pathological conditions (Pogue et al., 2010). On the other hand, miR-126 and miR-214 induce angiogenesis (Sheldon et al., 2010; van Balkom et al., 2013, 2015; Sharghi-Namini et al., 2014), while miR-296 increase levels of pro-angiogenic receptors (Würdinger et al., 2008). Several CNS disorders associated with defective neurogenesis has been linked to miRNAs function. For example, dysregulation of miR-124 is associated with neurodegenerative and stress-related disorders (Sun et al., 2015). Similarly, miR-155 is essential for inflammation-induced hippocampal neurogenic dysfunction via microglial activation (Woodbury et al., 2015).

Despite their relevant and determinant role on the neurogenic process, the precise target cells and mechanisms by which miRNAs are transferred to target cells in the neurogenic niche are virtually unknown. However, all the miRNAs mentioned above have been found in exosomes from different cellular origins (Table 1). Furthermore, most of them are expressed by different niche cells; thus, it is conceivable that some of these miRNA are transported within exosomes to exert their function on NSCs/NPCs and regulate the neurogenic process. In addition, a recent systemic characterization of exosomal RNA profiles in human plasma samples by RNA sequencing analyses showed that miRNAs were the most abundant (Huang et al., 2013). Interestingly, the same study showed that five (miR-128, miR-124, miR-125b, miR-9, and miR-let7b) out of the twenty most abundant exosomal miRNAs are involved in the neurogenic/angiogenic process. Thus, these results highlight not only the fact the neurogenesis-modulating miRNAs are incorporated into exosomes, but also stress the possibility of exosome-mediated communication between the systemic circulation and the CNS.

### Proteins and Signaling Peptides

Some of the biomolecules that have already been functionally characterized as modulators of adult neurogenesis, have also been described as components of exosomes. For example, TGF-β, a negative regulator of the adult neurogenic niche, has been documented to be present in exosomes of diverse cellular origins. As such, TGF-β-carrying exosomes circulate in the blood stream under diverse pathological conditions ranging from renal to pregnancy-related diseases and in consequence, might indirectly affect neurogenesis if crossing the BBB (Szajnik et al., 2013; Hong et al., 2014; Tan et al., 2014; Torreggiani et al., 2014; Raimondo et al., 2015; Solé et al., 2015). In addition to the protein, the mRNA coding for TGF-β has been found in exosomes from glioblastoma multiforme patients (Muller et al., 2015). On the other hand, positive regulators of adult neurogenesis such as Ephrin-B2 or components able to activate EGFR signaling have also been found in exosomes (Mathivanan et al., 2010; Higginbotham et al., 2011). Similarly, VEGF has been found in exosomes derived from several cell types (Thompson et al., 2013; Ekström et al., 2014; Torreggiani et al., 2014). Interestingly, it has been recently shown that adult hippocampal NSCs/NPCs secrete large amounts of VEGF *in vitro* and *in vivo* and this self-derived VEGF is functionally relevant for maintaining the neurogenic niche (Kirby et al., 2015). Several other signaling peptides known to determine cell fate in the neurogenic niche and described in the present work, such as PEDF, IGFBP6, EGF, FGF-2, Hedgehog, Notch, as well as proteins of the Wnt signaling pathway, just to mention a few, have been found in the exosomal fractions from different cell types and conditions (Graner et al., 2009; Nazarenko et al., 2010; Lai et al., 2011; Hajrasouliha et al., 2013; Wendler et al., 2013). Remarkably, most of the studies describing the presence of these proteins in exosomes correspond to proteomic analyses. In this regard, several intriguing issues remain to be addressed. The mechanisms involved in the delivery of several growth factors and signaling peptides to MVBs and their incorporation into ILVs are still speculative. Moreover, the molecular machinery responsible of transferring exosomal cargo into target cells are unknown. Further studies conducted to elucidate these molecular mechanisms will give clues about how adult neurogenesis is regulated not only under physiological conditions but also under certain CNS disorders.

## CNS Disorders Associated to Impaired Adult Neurogenesis: Potential Role of Exosomes in their Pathogenesis and as Biomarkers

Several neurodegenerative disorders have been associated to defects in the adult neurogenic process in the DG and/or subventricular zone/OB system (Steiner et al., 2006; Shruster et al., 2010; Mu and Gage, 2011; Winner et al., 2011; Regensburger et al., 2014; Foltynie, 2015; He and Nakayama, 2015; Le Grand et al., 2015; Winner and Winkler, 2015). On the other hand, significant progress has been done to unravel the function and regulation of adult neurogenesis in psychiatric diseases (Eisch et al., 2008; Jun et al., 2012). Depression is associated to a reduction in SGZ neural progenitor proliferation in the DG (Gould et al., 1998; Jacobs et al., 2000). Similarly, experimentally-induced inhibition of neurogenesis favors depressive-like behaviors in animal models (Wang et al., 2015; Xiang et al., 2015). On the other hand, different therapies that relieve depressive symptoms, such as antidepressant drug treatments (Malberg et al., 2000; Santarelli et al., 2003) or physical exercise (van Praag et al., 1999a; Lugert et al., 2010), increase SGZ neurogenesis. However, the mechanisms that stimulate adult hippocampal neurogenesis in those treatments are currently not clear (Jun et al., 2012).

Even though we are yet to obtain a conclusive causal relationship between adult neurogenesis and neurodegenerative or psychiatric diseases, growing evidence reveals how these disorders may proceed through impairment in the regulation of adult neurogenic niches. Thereby, despite their particular genetic or environmental origin, defects in adult neurogenesis appears as common hallmark functionally associated to the pathogenesis of different CNS diseases. Recent studies emphasize a putative role of exosomes in the pathogenesis of neurodegenerative and psychiatric disorders. Furthermore, considering that (i) exosomes may condition the microenvironment of a particular region such as the neurogenic niche; (ii) the content of exosomes in a particular cell type may change in different physiological and pathological conditions; and (iii) that brain parenchyma-derived exosomes may have access to the CSF and the peripheral blood circulation, neurogenic niche-derived exosomes may represent not only powerful regulators of adult neurogenesis, but also attractive therapeutic targets and useful biomarkers for different CNS disorders.

### Exosomes in Neurodegenerative Disorders

In neurodegenerative disorders such as Alzheimer’s disease (Rajendran et al., 2006; Saman et al., 2012), Parkinson’s disease (Emmanouilidou et al., 2010; Alvarez-Erviti et al., 2011a,b) and Prion diseases (Fevrier et al., 2004), the content of pathological forms of the toxic proteins associated to exosomes is increased and as such, it has been proposed that exosomes may favor the amplification and spread of protein misfolding diseases (Bellingham et al., 2012b; Danzer et al., 2012; Grad et al., 2014). In fact, when the production of exosomes was inhibited in a mouse model of Alzheimer’s disease by decreasing the synthesis of ceramide, a lipid enriched in exosomes, total amyloid as well as plaque levels were reduced (Dinkins et al., 2014). In brain cells, the abnormal protein aggregates present in neurodegenerative disorders activate the proteasome and autophagy pathways, tending to restore proteostasis (protein homeostasis; Hetz and Mollereau, 2014). A possible explanation of exosomal loading and secretion of cytotoxic proteins is that the overload of misfolded proteins may saturate the mentioned cytoprotective pathways, leading to their elimination via exosomes, although the mechanistic links of these processes are yet unknown (Baixauli et al., 2014). Interestingly, exosomes might also act by sequestering toxic proteins (Kalani et al., 2014). In Alzheimer’s Disease, in which exosomes are loaded with amyloid-β (Aβ) peptides and with molecules involved in its synthesis, degradation and aggregation, exogenous exosomes expressing surface proteins such as the cellular prion protein (PrPc), a receptor for Aβ, can sequester Aβ and counteract its negative effects on plasticity (An et al., 2013).

### Exosomes in Psychiatric Disorders

Almost nothing is known about changes in exosome content or release and their action on recipient cells in psychiatric disorders, such as schizophrenia, major depressive disorder, bipolar disorder or anxiety disorders, among others (Tsilioni et al., 2014). Interestingly, defects in the adult neurogenic process have been associated to the pathogenesis and/or progression of most of these disorders. In schizophrenia and bipolar disorders, specific exosome-related miRNAs (miR-497 and miR-29c, respectively) were upregulated in the prefrontal cortex of post-mortem brains (Banigan et al., 2013). We have shown that treatment with the antidepressant drug fluoxetine upregulates the content of the forebrain astrocyte-derived enzyme Aldolase C in CSF exosomes. The content of this enzyme is further upregulated in exosomes after chronic restraint stress, but not after stress induced by complete immobilization (Sandoval et al., 2013; Ampuero et al., 2015). A better knowledge of molecular changes associated to brain-derived exosomes in psychiatric disorders will allow a better comprehension of the neurobiological features of these complex diseases and in turn, would lead to the proposition of new treatment strategies.

### Exosomes as Potential Biomarkers of CNS Disorders

One of the yet unmet goals in CNS diseases, especially in the psychiatric sphere, is the establishment of biological markers, especially those obtained by non-invasive strategies, which could be used as diagnostic tools or to monitor disease progression, treatment effects and prognosis. Thus, the miRNA and protein cargo of exosomes obtained from peripheral body fluids, such as plasma or urine, constitute remarkable candidates as biomarkers (Cheng et al., 2014; Zhang et al., 2015a). With the exception of an intracranial cancer type, glioblastoma multiforme (Skog et al., 2008; Shao et al., 2012), it has not yet been possible to show in a convincing way that exosomes produced by neurons or glial cells might reach the blood circulation. Serum exosomes from glioblastoma multiforme patients carry tumor specific epidermal growth factor receptor vIII (EGFRvIII), and transforming growth factor beta 1 (TGF-β1; Skog et al., 2008; Graner et al., 2009). The proposed pathways are a defective BBB (Sáenz-Cuesta et al., 2014), a CSF-blood pathway or a transport process by transcytosis across endothelial cells in an intact BBB, as it has recently been suggested (Haqqani et al., 2013).

The fact that neurodegenerative diseases are associated with increases in plasma exosomes containing misfolded, pathological forms of proteins strongly suggest a central origin of them. Indeed, exosomes can carry proteins that serve as common biomarkers of a disease. A neuroblastoma cell line (SH-SY5Y) expressing α-synuclein release exosomes that contain this protein and are capable of transporting α-synuclein to SH-SY5Y cells that do not express it (Alvarez-Erviti et al., 2011a). *In vivo* experiments have shown that a small proportion of radioactively labeled α-synuclein delivered to the CSF can be recovered in plasma exosomes (Shi et al., 2014) pointing to a central origin of the protein, although its cellular origin remains obscure. Neurons infected with prion protein in its cellular (PrP^c^) as well as with its pathogenic form (PrP^sc^) release exosomes containing prion proteins as well as a specific miRNA signature (Fevrier et al., 2004; Vella et al., 2007; Bellingham et al., 2012a). In intracerebrally infected animals, exosomes carrying PrP^sc^, accounting for about 20% of the plasma infectivity, could be harvested from plasma samples (Properzi et al., 2015). In this study, PrP^sc^ was identified by Western blots with the caution of using sucrose gradient-purified exosomes to avoid the presence of blood-derived immunoglobulin contaminations that would obscure the usefulness of protein exosome markers in the molecular weight range of the IgG and IgM light and heavy chains (Properzi et al., 2015).

To identify EVs of neuronal origin, affinity purification using antibodies against neuronal membrane proteins, such as neural cell adhesion molecule-1 (NCAM-1) or neural cell adhesion molecule L1 (L1CAM), have been used. With this experimental strategy, the group of Kapogiannis has been able to detect plasma or serum exosomes with increased content of Aβ1–42, and of phosphorylated forms of tau and type 1 insulin receptor substrate (IRS-1) specifically associated to patients suffering Alzheimer’s Disease. Interestingly, these findings are proposed as useful predictive tools up to 10 years before the clinical onset of the disease (Fiandaca et al., 2015; Kapogiannis et al., 2015). In summary, the molecular content, i.e., miRNAs or proteins, in exosomes are emerging as strong candidates for providing CNS disease-specific biomarkers. The consistency of the results depends on the isolation method and thus, the purity of the fraction under analysis (exosomes vs. microparticles or microvesicles), or contamination with extravesicular blood-borne molecules while their origin in neuronal or glial cells is awaiting an indisputable proof. Furthermore, despite their purity, there are other shortcomings that need be to be addressed when attempting to use exosomes as biomarkers: (i) the pattern of exosomal RNA may change according to the extraction methods, due to differential susceptibility to exosomal lysis according to their membrane composition (Eldh et al., 2012; Van Aelst and Heymans, 2013); (ii) problems when trying to replicate previously published microarray analysis have been reported, mainly due to journals not enforcing more strict guidelines to interpret and report microarray data (Shields, 2006; Ioannidis et al., 2009); (iii) a further source of confusion is the possibility that medications taken by patients may alter the composition of exosomes, thus adding another level of complexity in the analysis and interpretation of the data (Aryani and Denecke, 2014).

## Conclusion and Future Perspectives

**Figure 4:**
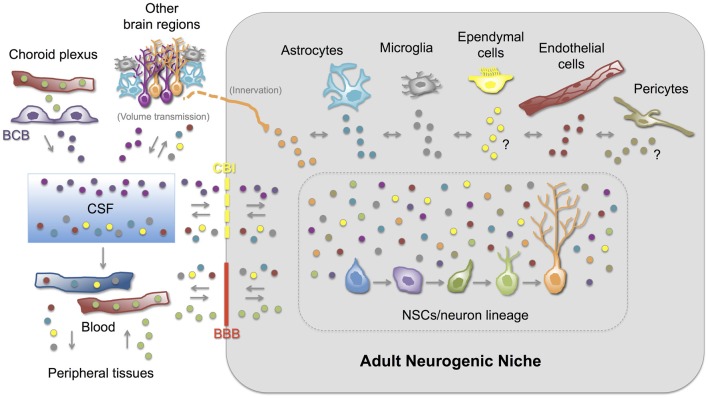
**Exosomes as regulators of adult neurogenesis.** The NSC-neuron lineage is exposed to a complex mix of exosomes within the neurogenic niche. Exosomes secreted locally by different niche cells can influence the physiology of other niche cells and the progression of different neurogenic stages. It is well demonstrated that neurons, astrocytes, microglia and endothelial cells secrete exosomes. NSCs/NPCs are also able to secrete exosomes (not depicted). Exosomes release by ependymal cells and pericytes has not been reported up to now (?). Additionally, exosomes originated in cells located far from the neurogenic niches can influence its nature. Exosomes derived from cells located in other brain regions can reach neurogenic niches through innervation of the niche or via CSF-mediated volume transmission. Indeed, besides being a source of soluble molecules for the SVZ neurogenic niche, the presence of exosomes in the CSF has been demonstrated in several mammalian species including humans. Furthermore, choroid plexus epithelial cells secrete exosomes into the CSF. Interestingly, exosomes produced in cells and tissues outside the central nervous system (CNS; peripheral tissues) can potentially reach neurogenic niches either directly, through the vasculature (blood) of the niche, or indirectly, through the choroid plexus. Conversely, exosomes originated in the neurogenic niche might have access to the CSF and to the peripheral blood circulation. The ability of exosomes to cross-communicate the CNS (neurogenic niches) with peripheral tissues highlights their potential role as physiological/pathological mediators of different CNS disorders (explanation for CNS-peripheral tissues co-morbidities, for example) and as biomarkers (CSF/blood samples). BBB, blood-brain barrier; BCB, blood-CSF barrier (choroid plexus epithelial cells); CBI, CSF-brain interface.

In this review, we focus on the potential role of EVs, and in particular, of exosomes in the regulation of the neurogenic niche (Figure 4). Exosomes, as messengers able to modulate the physiology of the niche, might originate from cells residing within the niche or from distant cells/tissues, thus having access to the neurogenic niche through the vasculature (blood-circulating exosomes) or by volume transmission via the CSF. The possible effects of molecular components already known to be present within exosomes on adult neurogenic process are also addressed. This scenario opens the possibility of a novel form of communication between niche cells and regulation of adult neurogenesis, able to both, regulate locally the extent of neurogenesis, and sense and integrate physiological conditions and pathological disturbances in diverse body systems.

On the other hand, exosomes originated in the CNS and in the neurogenic niche (i) can vary according to the circumstances, and (ii) might reach the peripheral blood circulation, thus linking or communicating directly the physiological or pathological status of the CNS to peripheral organs or tissues. This could be relevant (1) to understand the high prevalence of co-morbidity of pathologies associated to impaired neurogenesis and peripheral disorders, such as major depression and diabetes or inflammatory diseases (Kessler et al., 2003; Empana et al., 2005; Evans et al., 2005; Katon et al., 2008; Katon, 2008; Bonaz and Bernstein, 2013; Filipovic and Filipovic, 2014), and (2) to use blood-circulating CNS-derived exosomes as biomarkers of brain disorders.

The contribution of specific cell- and tissue-derived exosomes on adult neurogenesis should be further investigated with the use of proper animal models in which exosomes should be labeled with the use of molecular biology techniques, and later should be validated in health and disease with the use of a panel of biomarkers able to define specific exosome populations. We envisage that, in the near future, many of this work will be addressed by a growing community of researchers interested in the role of exosomes in disease-related processes that, among others, affect the neurogenic niche in a yet unsuspected manner.

## Author Contributions

All the authors have contributed substantially to the writing and revising of the manuscript. LFB and UW have participated in the conception and design of the work. LFB have designed the figures. RIM, ZDV and LFB have designed and completed the table. MAC, RIM, PVB, ZDV, CAL, P-ET have participated in writing, drafting and revising of different sections of the manuscript. All the authors have approved the final version of the manuscript.

## Conflict of Interest Statement

The authors declare that the research was conducted in the absence of any commercial or financial relationships that could be construed as a potential conflict of interest.

## Footnotes

^1^http://www.exocarta.org

^2^http://www.microvesicles.org/

^3^http://student4.postech.ac.kr/evpedia2_xe/xe/

## References

[B1] Abrous D. N. Koehl M. Le Moal M. (2005). Adult neurogenesis: from precursors to network and physiology. Physiol. Rev. 85, 523–569. 10.1152/physrev.00055.2003 15788705

[B2] Admyre C. Telemo E. Almqvist N. Lötvall J. Lahesmaa R. Scheynius A.. (2008). Exosomes—nanovesicles with possible roles in allergic inflammation. Allergy 63, 404–408. 10.1111/j.1398-9995.2007.01600.x 18315728

[B3] Agnati L. F. Fuxe K. (2014). Extracellular-vesicle type of volume transmission and tunnelling-nanotube type of wiring transmission add a new dimension to brain neuro-glial networks. Philos. Trans. R. Soc. Lond. B Biol. Sci. 369:20130505. 10.1098/rstb.2013.0505 25135966 PMC4142026

[B4] Aimone J. B. Li Y. Lee S. W. Clemenson G. D. Deng W. Gage F. H. (2014). Regulation and function of adult neurogenesis: from genes to cognition. Physiol. Rev. 94, 991–1026. 10.1152/physrev.00004.2014 25287858 PMC4280160

[B5] Akerblom M. Jakobsson J. (2013). MicroRNAs as neuronal fate determinants. Neuroscientist 20, 235–242. 10.1177/1073858413497265 23877999

[B6] Akerblom M. Sachdeva R. Barde I. Verp S. Gentner B. Trono D.. (2012). MicroRNA-124 is a subventricular zone neuronal fate determinant. J. Neurosci. 32, 8879–8889. 10.1523/JNEUROSCI.0558-12.2012 22745489 PMC4434222

[B7] Altman J. (1969). Autoradiographic and histological studies of postnatal neurogenesis. IV. Cell proliferation and migration in the anterior forebrain, with special reference to persisting neurogenesis in the olfactory bulb. J. Comp. Neurol. 137, 433–457. 10.1002/cne.901370404 5361244

[B8] Altman J. Das G. D. (1965). Post-natal origin of microneurones in the rat brain. Nature 207, 953–956. 10.1038/207953a0 5886931

[B9] Altman J. Das G. D. (1966). Autoradiographic and histological studies of postnatal neurogenesis. I. A longitudinal investigation of the kinetics, migration and transformation of cells incorporating tritiated thymidine in neonate rats, with special reference to postnatal neurogenesis in some brain regions. J. Comp. Neurol. 126, 337–389. 10.1002/cne.901260302 5937257

[B11] Alvarez-Buylla A. Kohwi M. Nguyen T. M. Merkle F. T. (2008). The heterogeneity of adult neural stem cells and the emerging complexity of their niche. Cold Spring Harb. Symp. Quant. Biol. 73, 357–365. 10.1101/sq11.2008.73.019 19022766

[B10] Alvarez-Buylla A. Lim D. A. (2004). For the long run: maintaining germinal niches in the adult brain. Neuron 41, 683–686. 10.1016/S0896-6273(04)00111-4 15003168

[B12] Alvarez-Erviti L. Couch Y. Richardson J. Cooper J. M. Wood M. J. (2011a). Alpha-synuclein release by neurons activates the inflammatory response in a microglial cell line. Neurosci. Res. 69, 337–342. 10.1016/j.neures.2010.12.020 21255620

[B13] Alvarez-Erviti L. Seow Y. Schapira A. H. Gardiner C. Sargent I. L. Wood M. J.. (2011b). Lysosomal dysfunction increases exosome-mediated alpha-synuclein release and transmission. Neurobiol. Dis. 42, 360–367. 10.1016/j.nbd.2011.01.029 21303699 PMC3107939

[B14] Alvarez-Erviti L. Seow Y. Yin H. Betts C. Lakhal S. Wood M. J. (2011c). Delivery of siRNA to the mouse brain by systemic injection of targeted exosomes. Nat. Biotechnol. 29, 341–345. 10.1038/nbt.1807 21423189

[B15] Ampuero E. Luarte A. Santibañez M. Varas-Godoy M. Toledo J. Diaz-Veliz G.. (2015). Two chronic stress models based on movement restriction in rats respond selectively to antidepressant drugs: aldolase c as a potential biomarker. Int. J. Neuropsychopharmacol. 18:pyv038. 10.1093/ijnp/pyv038 25813018 PMC4648154

[B16] An K. Klyubin I. Kim Y. Jung J. H. Mably A. J. O’Dowd S. T.. (2013). Exosomes neutralize synaptic-plasticity-disrupting activity of Abeta assemblies *in vivo*. Mol. Brain 6:47. 10.1186/1756-6606-6-47 24284042 PMC4222117

[B17] Andreu-Agullo C. Morante-Redolat J. M. Delgado A. C. Fariñas I. (2009). Vascular niche factor PEDF modulates Notch-dependent stemness in the adult subependymal zone. Nat. Neurosci. 12, 1514–1523. 10.1038/nn.2437 19898467

[B18] Armulik A. Genové G. Betsholtz C. (2011). Pericytes: developmental, physiological and pathological perspectives, problems and promises. Dev. Cell 21, 193–215. 10.1016/j.devcel.2011.07.001 21839917

[B19] Aryani A. Denecke B. (2014). Exosomes as a nanodelivery system: a key to the future of neuromedicine? Mol. Neurobiol. [Epub ahead of print]. 10.1007/s12035-014-9054-5 25502465 PMC4752585

[B20] Ashton R. S. Conway A. Pangarkar C. Bergen J. Lim K. I. Shah P.. (2012). Astrocytes regulate adult hippocampal neurogenesis through ephrin-B signaling. Nat. Neurosci. 15, 1399–1406. 10.1038/nn.3212 22983209 PMC3458152

[B21] Bahrini I. Song J. H. Diez D. Hanayama R. (2015). Neuronal exosomes facilitate synaptic pruning by up-regulating complement factors in microglia. Sci. Rep. 5:7989. 10.1038/srep07989 25612542 PMC4303875

[B22] Baietti M. F. Zhang Z. Mortier E. Melchior A. Degeest G. Geeraerts A.. (2012). Syndecan-syntenin-ALIX regulates the biogenesis of exosomes. Nat. Cell Biol. 14, 677–685. 10.1038/ncb2502 22660413

[B23] Baixauli F. Lopez-Otin C. Mittelbrunn M. (2014). Exosomes and autophagy: coordinated mechanisms for the maintenance of cellular fitness. Front. Immunol. 5:403. 10.3389/fimmu.2014.00403 25191326 PMC4138502

[B24] Baker S. A. Baker K. A. Hagg T. (2004). Dopaminergic nigrostriatal projections regulate neural precursor proliferation in the adult mouse subventricular zone. Eur. J. Neurosci. 20, 575–579. 10.1111/j.1460-9568.2004.03486.x 15233767

[B25] Banigan M. G. Kao P. F. Kozubek J. A. Winslow A. R. Medina J. Costa J.. (2013). Differential expression of exosomal microRNAs in prefrontal cortices of schizophrenia and bipolar disorder patients. PLoS One 8:e48814. 10.1371/journal.pone.0048814 23382797 PMC3559697

[B26] Banizs B. Pike M. M. Millican C. L. Ferguson W. B. Komlosi P. Sheetz J.. (2005). Dysfunctional cilia lead to altered ependyma and choroid plexus function and result in the formation of hydrocephalus. Development 132, 5329–5339. 10.1242/dev.02153 16284123

[B27] Barkho B. Z. Song H. Aimone J. B. Smrt R. D. Kuwabara T. Nakashima K.. (2006). Identification of astrocyte-expressed factors that modulate neural stem/progenitor cell differentiation. Stem Cells Dev. 15, 407–421. 10.1089/scd.2006.15.407 16846377 PMC2777811

[B28] Baruch K. Deczkowska A. David E. Castellano J. M. Miller O. Kertser A.. (2014). Aging. Aging-induced type I interferon response at the choroid plexus negatively affects brain function. Science 346, 89–93. 10.1126/science.1252945 25147279 PMC4869326

[B29] Bátiz L. F. Jiménez A. J. Guerra M. Rodríguez-Pérez L. M. Toledo C. D. Vio K.. (2011). New ependymal cells are born postnatally in two discrete regions of the mouse brain and support ventricular enlargement in hydrocephalus. Acta Neuropathol. 121, 721–735. 10.1007/s00401-011-0799-x 21311902

[B30] Battista D. Ferrari C. C. Gage F. H. Pitossi F. J. (2006). Neurogenic niche modulation by activated microglia: transforming growth factor beta increases neurogenesis in the adult dentate gyrus. Eur. J. Neurosci. 23, 83–93. 10.1111/j.1460-9568.2005.04539.x 16420418

[B31] Bellingham S. A. Coleman B. M. Hill A. F. (2012a). Small RNA deep sequencing reveals a distinct miRNA signature released in exosomes from prion-infected neuronal cells. Nucleic Acids Res. 40, 10937–10949. 10.1093/nar/gks832 22965126 PMC3505968

[B32] Bellingham S. A. Guo B. B. Coleman B. M. Hill A. F. (2012b). Exosomes: vehicles for the transfer of toxic proteins associated with neurodegenerative diseases? Front. Physiol. 3:124. 10.3389/fphys.2012.00124 22563321 PMC3342525

[B33] Berg D. A. Belnoue L. Song H. Simon A. (2013). Neurotransmitter-mediated control of neurogenesis in the adult vertebrate brain. Development 140, 2548–2561. 10.1242/dev.088005 23715548 PMC3666382

[B34] Bergmann O. Spalding K. L. Frisén J. (2015). Adult neurogenesis in humans. Cold Spring Harb. Perspect. Biol. 7:a018994. 10.1101/cshperspect.a018994 26134318 PMC4484963

[B35] Bian S. Xu T. L. Sun T. (2013). Tuning the cell fate of neurons and glia by microRNAs. Curr. Opin. Neurobiol. 23, 928–934. 10.1016/j.conb.2013.08.002 23978589 PMC3830639

[B36] Bianco F. Perrotta C. Novellino L. Francolini M. Riganti L. Menna E.. (2009). Acid sphingomyelinase activity triggers microparticle release from glial cells. EMBO J. 28, 1043–1054. 10.1038/emboj.2009.45 19300439 PMC2664656

[B37] Bianco F. Pravettoni E. Colombo A. Schenk U. Möller T. Matteoli M.. (2005). Astrocyte-derived ATP induces vesicle shedding and IL-1 beta release from microglia. J. Immunol. 174, 7268–7277. 10.4049/jimmunol.174.11.7268 15905573

[B38] Bjornsson C. S. Apostolopoulou M. Tian Y. Temple S. (2015). It takes a village: constructing the neurogenic niche. Dev. Cell 32, 435–446. 10.1016/j.devcel.2015.01.010 25710530 PMC4886554

[B39] Bobrie A. Colombo M. Raposo G. Théry C. (2011). Exosome secretion: molecular mechanisms and roles in immune responses. Traffic 12, 1659–1668. 10.1111/j.1600-0854.2011.01225.x 21645191

[B40] Bolteus A. J. Bordey A. (2004). GABA release and uptake regulate neuronal precursor migration in the postnatal subventricular zone. J. Neurosci. 24, 7623–7631. 10.1523/jneurosci.1999-04.2004 15342728 PMC6729616

[B41] Bonaz B. L. Bernstein C. N. (2013). Brain-gut interactions in inflammatory bowel disease. Gastroenterology 144, 36–49. 10.1053/j.gastro.2012.10.003 23063970

[B42] Borroto-Escuela D. O. Agnati L. F. Bechter K. Jansson A. Tarakanov A. O. Fuxe K. (2015). The role of transmitter diffusion and flow versus extracellular vesicles in volume transmission in the brain neural-glial networks. Philos. Trans. R. Soc. Lond. B Biol. Sci. 370:20140183. 10.1098/rstb.2014.0183 26009762 PMC4455752

[B43] Bozoyan L. Khlghatyan J. Saghatelyan A. (2012). Astrocytes control the development of the migration-promoting vasculature scaffold in the postnatal brain via VEGF signaling. J. Neurosci. 32, 1687–1704. 10.1523/JNEUROSCI.5531-11.2012 22302810 PMC6703370

[B44] Brightman M. W. Reese T. S. (1969). Junctions between intimately apposed cell membranes in the vertebrate brain. J. Cell Biol. 40, 648–677. 10.1083/jcb.40.3.648 5765759 PMC2107650

[B45] Brites D. Vaz A. R. (2014). Microglia centered pathogenesis in ALS: insights in cell interconnectivity. Front. Cell. Neurosci. 8:117. 10.3389/fncel.2014.00117 24904276 PMC4033073

[B46] Bunn R. C. King W. D. Winkler M. K. Fowlkes J. L. (2005). Early developmental changes in IGF-I, IGF-II, IGF binding protein-1 and IGF binding protein-3 concentration in the cerebrospinal fluid of children. Pediatr. Res. 58, 89–93. 10.1203/01.pdr.0000156512.27710.a9 15774848

[B47] Butovsky O. Koronyo-Hamaoui M. Kunis G. Ophir E. Landa G. Cohen H.. (2006). Glatiramer acetate fights against Alzheimer’s disease by inducing dendritic-like microglia expressing insulin-like growth factor 1. Proc. Natl. Acad. Sci. U S A 103, 11784–11789. 10.1073/pnas.0604681103 16864778 PMC1544247

[B48] Cameron H. A. Dayer A. G. (2008). New interneurons in the adult neocortex: small, sparse, but significant? Biol. Psychiatry 63, 650–655. 10.1016/j.biopsych.2007.09.023 18067877 PMC2423203

[B49] Cao L. Jiao X. Zuzga D. S. Liu Y. Fong D. M. Young D.. (2004). VEGF links hippocampal activity with neurogenesis, learning and memory. Nat. Genet. 36, 827–835. 10.1038/ng1395 15258583

[B50] Cao X. Li L. P. Qin X. H. Li S. J. Zhang M. Wang Q.. (2013). Astrocytic adenosine 5’-triphosphate release regulates the proliferation of neural stem cells in the adult hippocampus. Stem Cells 31, 1633–1643. 10.1002/stem.1408 23630193

[B51] Carlén M. Meletis K. Göritz C. Darsalia V. Evergren E. Tanigaki K.. (2009). Forebrain ependymal cells are Notch-dependent and generate neuroblasts and astrocytes after stroke. Nat. Neurosci. 12, 259–267. 10.1038/nn.2268 19234458

[B52] Carpentier P. A. Palmer T. D. (2009). Immune influence on adult neural stem cell regulation and function. Neuron 64, 79–92. 10.1016/j.neuron.2009.08.038 19840551 PMC2789107

[B53] Cernilogar F. M. Di Giaimo R. Rehfeld F. Cappello S. Lie D. C. (2015). RNA interference machinery-mediated gene regulation in mouse adult neural stem cells. BMC Neurosci. 16:60. 10.1186/s12868-015-0198-7 26386671 PMC4575781

[B54] Chaput N. Théry C. (2011). Exosomes: immune properties and potential clinical implementations. Semin. Immunopathol. 33, 419–440. 10.1007/s00281-010-0233-9 21174094

[B56] Cheng L. C. Pastrana E. Tavazoie M. Doetsch F. (2009). miR-124 regulates adult neurogenesis in the subventricular zone stem cell niche. Nat. Neurosci. 12, 399–408. 10.1038/nn.2294 19287386 PMC2766245

[B55] Cheng L. Sharples R. A. Scicluna B. J. Hill A. F. (2014). Exosomes provide a protective and enriched source of miRNA for biomarker profiling compared to intracellular and cell-free blood. J. Extracell. Vesicles 3:23743. 10.3402/jev.v3.23743 24683445 PMC3968297

[B57] Chiasserini D. van Weering J. R. Piersma S. R. Pham T. V. Malekzadeh A. Teunissen C. E.. (2014). Proteomic analysis of cerebrospinal fluid extracellular vesicles: a comprehensive dataset. J. Proteomics 106, 191–204. 10.1016/j.jprot.2014.04.028 24769233

[B58] Chivet M. Hemming F. Pernet-Gallay K. Fraboulet S. Sadoul R. (2012). Emerging role of neuronal exosomes in the central nervous system. Front. Physiol. 3:145. 10.3389/fphys.2012.00145 22654762 PMC3361079

[B59] Chivet M. Javalet C. Hemming F. Pernet-Gallay K. Laulagnier K. Fraboulet S.. (2013). Exosomes as a novel way of interneuronal communication. Biochem. Soc. Trans. 41, 241–244. 10.1042/BST20120266 23356290

[B60] Chivet M. Javalet C. Laulagnier K. Blot B. Hemming F. J. Sadoul R. (2014). Exosomes secreted by cortical neurons upon glutamatergic synapse activation specifically interact with neurons. J. Extracell. Vesicles 3:24722. 10.3402/jev.v3.24722 25398455 PMC4232649

[B61] Chojnacki A. K. Mak G. K. Weiss S. (2009). Identity crisis for adult periventricular neural stem cells: subventricular zone astrocytes, ependymal cells or both? Nat. Rev. Neurosci. 10, 153–163. 10.1038/nrn2571 19153578

[B62] Cocucci E. Meldolesi J. (2015). Ectosomes and exosomes: shedding the confusion between extracellular vesicles. Trends Cell Biol. 25, 364–372. 10.1016/j.tcb.2015.01.004 25683921

[B63] Codega P. Silva-Vargas V. Paul A. Maldonado-Soto A. R. Deleo A. M. Pastrana E.. (2014). Prospective identification and purification of quiescent adult neural stem cells from their *in vivo* niche. Neuron 82, 545–559. 10.1016/j.neuron.2014.02.039 24811379 PMC4360885

[B64] Colak D. Mori T. Brill M. S. Pfeifer A. Falk S. Deng C.. (2008). Adult neurogenesis requires Smad4-mediated bone morphogenic protein signaling in stem cells. J. Neurosci. 28, 434–446. 10.1523/JNEUROSCI.4374-07.2008 18184786 PMC6670509

[B65] Colombo M. Raposo G. Théry C. (2014). Biogenesis, secretion and intercellular interactions of exosomes and other extracellular vesicles. Annu. Rev. Cell Dev. Biol. 30, 255–289. 10.1146/annurev-cellbio-101512-122326 25288114

[B66] Conover J. C. Notti R. Q. (2008). The neural stem cell niche. Cell Tissue Res. 331, 211–224. 10.1007/s00441-007-0503-6 17922142

[B67] Cooper J. M. Wiklander P. B. Nordin J. Z. Al-Shawi R. Wood M. J. Vithlani M.. (2014). Systemic exosomal siRNA delivery reduced alpha-synuclein aggregates in brains of transgenic mice. Mov. Disord. 29, 1476–1485. 10.1002/mds.25978 25112864 PMC4204174

[B68] Cossetti C. Iraci N. Mercer T. R. Leonardi T. Alpi E. Drago D.. (2014). Extracellular vesicles from neural stem cells transfer IFN-gamma via Ifngr1 to activate Stat1 signaling in target cells. Mol. Cell 56, 193–204. 10.1016/j.molcel.2014.08.020 25242146 PMC4578249

[B69] Costa-Silva B. Aiello N. M. Ocean A. J. Singh S. Zhang H. Thakur B. K.. (2015). Pancreatic cancer exosomes initiate pre-metastatic niche formation in the liver. Nat. Cell Biol. 17, 816–826. 10.1038/ncb3169 25985394 PMC5769922

[B70] Crouch E. E. Liu C. Silva-Vargas V. Doetsch F. (2015). Regional and stage-specific effects of prospectively purified vascular cells on the adult V-SVZ neural stem cell lineage. J. Neurosci. 35, 4528–4539. 10.1523/JNEUROSCI.1188-14.2015 25788671 PMC4363382

[B71] Curtis M. A. Kam M. Nannmark U. Anderson M. F. Axell M. Z. Wikkelso C.. (2007). Human neuroblasts migrate to the olfactory bulb via a lateral ventricular extension. Science 315, 1243–1249. 10.1126/science.1136281 17303719

[B72] Danzer K. M. Kranich L. R. Ruf W. P. Cagsal-Getkin O. Winslow A. R. Zhu L.. (2012). Exosomal cell-to-cell transmission of alpha synuclein oligomers. Mol. Neurodegener. 7:42. 10.1186/1750-1326-7-42 22920859 PMC3483256

[B73] de Jong O. G. Verhaar M. C. Chen Y. Vader P. Gremmels H. Posthuma G.. (2012). Cellular stress conditions are reflected in the protein and RNA content of endothelial cell-derived exosomes. J. Extracell. Vesicles 1:18396. 10.3402/jev.v1i0.18396 24009886 PMC3760650

[B74] Del Bigio M. R. (1995). The ependyma: a protective barrier between brain and cerebrospinal fluid. Glia 14, 1–13. 10.1002/glia.440140102 7615341

[B75] Delgado A. C. Ferrón S. R. Vicente D. Porlan E. Perez-Villalba A. Trujillo C. M.. (2014). Endothelial NT-3 delivered by vasculature and CSF promotes quiescence of subependymal neural stem cells through nitric oxide induction. Neuron 83, 572–585. 10.1016/j.neuron.2014.06.015 25043422

[B76] Dinkins M. B. Dasgupta S. Wang G. Zhu G. Bieberich E. (2014). Exosome reduction *in vivo* is associated with lower amyloid plaque load in the 5XFAD mouse model of Alzheimer’s disease. Neurobiol. Aging 35, 1792–1800. 10.1016/j.neurobiolaging.2014.02.012 24650793 PMC4035236

[B77] Doetsch F. Garcia-Verdugo J. M. Alvarez-Buylla A. (1997). Cellular composition and three-dimensional organization of the subventricular germinal zone in the adult mammalian brain. J. Neurosci. 17, 5046–5061. 9185542 10.1523/JNEUROSCI.17-13-05046.1997 PMC6573289

[B78] Dohgu S. Takata F. Yamauchi A. Nakagawa S. Egawa T. Naito M.. (2005). Brain pericytes contribute to the induction and up-regulation of blood-brain barrier functions through transforming growth factor-beta production. Brain Res. 1038, 208–215. 10.1016/j.brainres.2005.01.027 15757636

[B79] Domínguez-Pinos M. D. Páez P. Jiménez A. J. Weil B. Arráez M. A. Pérez-Fígares J. M.. (2005). Ependymal denudation and alterations of the subventricular zone occur in human fetuses with a moderate communicating hydrocephalus. J. Neuropathol. Exp. Neurol. 64, 595–604. 10.1097/01.jnen.0000171648.86718.bb 16042311

[B80] Dore-Duffy P. Cleary K. (2011). Morphology and properties of pericytes. Methods Mol. Biol. 686, 49–68. 10.1007/978-1-60761-938-3_2 21082366

[B81] Dore-Duffy P. Katychev A. Wang X. Van Buren E. (2006). CNS microvascular pericytes exhibit multipotential stem cell activity. J. Cereb. Blood Flow Metab. 26, 613–624. 10.1038/sj.jcbfm.9600272 16421511

[B82] Douglas-Akinwande A. C. Buckwalter K. A. Rydberg J. Rankin J. L. Choplin R. H. (2006). Multichannel CT: evaluating the spine in postoperative patients with orthopedic hardware. Radiographics 26, S97–S110. 10.1148/rg.26si065512 17050522

[B83] Drago D. Cossetti C. Iraci N. Gaude E. Musco G. Bachi A.. (2013). The stem cell secretome and its role in brain repair. Biochimie 95, 2271–2285. 10.1016/j.biochi.2013.06.020 23827856 PMC4061727

[B84] Edgar B. A. Zielke N. Gutierrez C. (2014). Endocycles: a recurrent evolutionary innovation for post-mitotic cell growth. Nat. Rev. Mol. Cell Biol. 15, 197–210. 10.1038/nrm3756 24556841

[B85] Egeland M. Zunszain P. A. Pariante C. M. (2015). Molecular mechanisms in the regulation of adult neurogenesis during stress. Nat. Rev. Neurosci. 16, 189–200. 10.1038/nrn3855 25790864

[B86] Eisch A. J. Cameron H. A. Encinas J. M. Meltzer L. A. Ming G. L. Overstreet-Wadiche L. S. (2008). Adult neurogenesis, mental health and mental illness: hope or hype? J. Neurosci. 28, 11785–11791. 10.1523/JNEUROSCI.3798-08.2008 19005040 PMC2793333

[B87] Ekström E. J. Bergenfelz C. von Bülow V. Serifler F. Carlemalm E. Jönsson G.. (2014). WNT5A induces release of exosomes containing pro-angiogenic and immunosuppressive factors from malignant melanoma cells. Mol. Cancer 13:88. 10.1186/1476-4598-13-88 24766647 PMC4022450

[B88] El-Andaloussi S. Lee Y. Lakhal-Littleton S. Li J. Seow Y. Gardiner C.. (2012). Exosome-mediated delivery of siRNA *in vitro* and *in vivo*. Nat. Protoc. 7, 2112–2126. 10.1038/nprot.2012.131 23154783

[B89] Eldh M. Lötvall J. Malmhüll C. Ekström K. (2012). Importance of RNA isolation methods for analysis of exosomal RNA: evaluation of different methods. Mol. Immunol. 50, 278–286. 10.1016/j.molimm.2012.02.001 22424315

[B90] Emmanouilidou E. Melachroinou K. Roumeliotis T. Garbis S. D. Ntzouni M. Margaritis L. H.. (2010). Cell-produced alpha-synuclein is secreted in a calcium-dependent manner by exosomes and impacts neuronal survival. J. Neurosci. 30, 6838–6851. 10.1523/JNEUROSCI.5699-09.2010 20484626 PMC3842464

[B91] Empana J. P. Sykes D. H. Luc G. Juhan-Vague I. Arveiler D. Ferrieres J.. (2005). Contributions of depressive mood and circulating inflammatory markers to coronary heart disease in healthy European men: the Prospective Epidemiological Study of Myocardial Infarction (PRIME). Circulation 111, 2299–2305. 10.1161/01.cir.0000164203.54111.ae 15867179

[B92] Engelhardt B. Sorokin L. (2009). The blood-brain and the blood-cerebrospinal fluid barriers: function and dysfunction. Semin. Immunopathol. 31, 497–511. 10.1007/s00281-009-0177-0 19779720

[B93] Eriksson P. S. Perfilieva E. Björk-Eriksson T. Alborn A. M. Nordborg C. Peterson D. A.. (1998). Neurogenesis in the adult human hippocampus. Nat. Med. 4, 1313–1317. 10.1038/3305 9809557

[B94] Escudero C. A. Lazo O. M. Galleguillos C. Parraguez J. I. Lopez-Verrilli M. A. Cabeza C.. (2014). The p75 neurotrophin receptor evades the endolysosomal route in neuronal cells, favouring multivesicular bodies specialised for exosomal release. J. Cell Sci. 127, 1966–1979. 10.1242/jcs.141754 24569882 PMC4004974

[B95] Evans D. L. Charney D. S. Lewis L. Golden R. N. Gorman J. M. Krishnan K. R.. (2005). Mood disorders in the medically ill: scientific review and recommendations. Biol. Psychiatry 58, 175–189. 10.1016/j.biopsych.2005.05.001 16084838

[B96] Fabel K. Fabel K. Tam B. Kaufer D. Baiker A. Simmons N.. (2003). VEGF is necessary for exercise-induced adult hippocampal neurogenesis. Eur. J. Neurosci. 18, 2803–2812. 10.1111/j.1460-9568.2003.03041.x 14656329

[B97] Faure J. Lachenal G. Court M. Hirrlinger J. Chatellard-Causse C. Blot B.. (2006). Exosomes are released by cultured cortical neurones. Mol. Cell. Neurosci. 31, 642–648. 10.1016/j.mcn.2005.12.003 16446100

[B98] Fawcett J. W. Asher R. A. (1999). The glial scar and central nervous system repair. Brain Res. Bull. 49, 377–391. 10.1016/s0361-9230(99)00072-6 10483914

[B99] Feliciano D. M. Zhang S. Nasrallah C. M. Lisgo S. N. Bordey A. (2014). Embryonic cerebrospinal fluid nanovesicles carry evolutionarily conserved molecules and promote neural stem cell amplification. PLoS One 9:e88810. 10.1371/journal.pone.0088810 24533152 PMC3923048

[B100] Ferland R. J. Bátiz L. F. Neal J. Lian G. Bundock E. Lu J.. (2009). Disruption of neural progenitors along the ventricular and subventricular zones in periventricular heterotopia. Hum. Mol. Genet. 18, 497–516. 10.1093/hmg/ddn377 18996916 PMC2722192

[B101] Fevrier B. Vilette D. Archer F. Loew D. Faigle W. Vidal M.. (2004). Cells release prions in association with exosomes. Proc. Natl. Acad. Sci. U S A 101, 9683–9688. 10.1073/pnas.0308413101 15210972 PMC470735

[B102] Fiandaca M. S. Kapogiannis D. Mapstone M. Boxer A. Eitan E. Schwartz J. B.. (2015). Identification of preclinical Alzheimer’s disease by a profile of pathogenic proteins in neurally derived blood exosomes: a case-control study. Alzheimers Dement. 11, 600.e1–607.e1. 10.1016/j.jalz.2014.06.008 25130657 PMC4329112

[B103] Filipovic B. R. Filipovic B. F. (2014). Psychiatric comorbidity in the treatment of patients with inflammatory bowel disease. World J. Gastroenterol. 20, 3552–3563. 10.3748/wjg.v20.i13.3552 24707138 PMC3974522

[B104] Fitzner D. Schnaars M. van Rossum D. Krishnamoorthy G. Dibaj P. Bakhti M.. (2011). Selective transfer of exosomes from oligodendrocytes to microglia by macropinocytosis. J. Cell Sci. 124, 447–458. 10.1242/jcs.074088 21242314

[B105] Foltynie T. (2015). Can Parkinson’s disease be cured by stimulating neurogenesis? J. Clin. Invest. 125, 978–980. 10.1172/JCI80822 25689259 PMC4362270

[B106] Fröhlich D. Kuo W. P. Frühbeis C. Sun J. J. Zehendner C. M. Luhmann H. J.. (2014). Multifaceted effects of oligodendroglial exosomes on neurons: impact on neuronal firing rate, signal transduction and gene regulation. Philos. Trans. R. Soc. Lond. B Biol. Sci. 369:20130510. 10.1098/rstb.2013.0510 25135971 PMC4142031

[B107] Frühbeis C. Fröhlich D. Krämer-Albers E. M. (2012). Emerging roles of exosomes in neuron-glia communication. Front. Physiol. 3:119. 10.3389/fphys.2012.00119 22557979 PMC3339323

[B108] Frühbeis C. Fröhlich D. Kuo W. P. Amphornrat J. Thilemann S. Saab A. S.. (2013a). Neurotransmitter-triggered transfer of exosomes mediates oligodendrocyte-neuron communication. PLoS Biol. 11:e1001604. 10.1371/journal.pbio.1001604 23874151 PMC3706306

[B109] Frühbeis C. Fröhlich D. Kuo W. P. Krämer-Albers E. M. (2013b). Extracellular vesicles as mediators of neuron-glia communication. Front. Cell. Neurosci. 7:182. 10.3389/fncel.2013.00182 24194697 PMC3812991

[B110] Gage F. H. (2000). Mammalian neural stem cells. Science 287, 1433–1438. 10.1126/science.287.5457.1433 10688783

[B111] Ge S. Goh E. L. Sailor K. A. Kitabatake Y. Ming G. L. Song H. (2006). GABA regulates synaptic integration of newly generated neurons in the adult brain. Nature 439, 589–593. 10.1038/nature04404 16341203 PMC1420640

[B112] Gebara E. Sultan S. Kocher-Braissant J. Toni N. (2013). Adult hippocampal neurogenesis inversely correlates with microglia in conditions of voluntary running and aging. Front. Neurosci. 7:145. 10.3389/fnins.2013.00145 23970848 PMC3747329

[B113] Gemma C. Bachstetter A. D. Bickford P. C. (2010). Neuron-microglia dialogue and hippocampal neurogenesis in the aged brain. Aging Dis. 1, 232–244. 21961084 PMC3180926

[B114] Ghidoni R. Paterlini A. Albertini V. Glionna M. Monti E. Schiaffonati L.. (2011). Cystatin C is released in association with exosomes: a new tool of neuronal communication which is unbalanced in Alzheimer’s disease. Neurobiol. Aging 32, 1435–1442. 10.1016/j.neurobiolaging.2009.08.013 19773092 PMC2891183

[B115] Ginhoux F. Lim S. Hoeffel G. Low D. Huber T. (2013). Origin and differentiation of microglia. Front. Cell. Neurosci. 7:45. 10.3389/fncel.2013.00045 23616747 PMC3627983

[B116] Girard F. Eichenberger S. Celio M. R. (2014). Thrombospondin 4 deficiency in mouse impairs neuronal migration in the early postnatal and adult brain. Mol. Cell. Neurosci. 61, 176–186. 10.1016/j.mcn.2014.06.010 24983516

[B117] Glebov K. Löchner M. Jabs R. Lau T. Merkel O. Schloss P.. (2015). Serotonin stimulates secretion of exosomes from microglia cells. Glia 63, 626–634. 10.1002/glia.22772 25451814

[B118] Gomez-Gaviro M. V. Scott C. E. Sesay A. K. Matheu A. Booth S. Galichet C.. (2012). Betacellulin promotes cell proliferation in the neural stem cell niche and stimulates neurogenesis. Proc. Natl. Acad. Sci. U S A 109, 1317–1322. 10.1073/pnas.1016199109 22232668 PMC3268286

[B119] Gomez-Nicola D. Perry V. H. (2015). Microglial dynamics and role in the healthy and diseased brain: a paradigm of functional plasticity. Neuroscientist 21, 169–184. 10.1177/1073858414530512 24722525 PMC4412879

[B120] Gould E. (2007). How widespread is adult neurogenesis in mammals? Nat. Rev. Neurosci. 8, 481–488. 10.1038/nrn2147 17514200

[B123] Gould G. W. Lippincott-Schwartz J. (2009). New roles for endosomes: from vesicular carriers to multi-purpose platforms. Nat. Rev. Mol. Cell Biol. 10, 287–292. 10.1038/nrm2652 19277045 PMC3690957

[B121] Gould E. Reeves A. J. Graziano M. S. Gross C. G. (1999). Neurogenesis in the neocortex of adult primates. Science 286, 548–552. 10.1126/science.286.5439.548 10521353

[B122] Gould E. Tanapat P. McEwen B. S. Flügge G. Fuchs E. (1998). Proliferation of granule cell precursors in the dentate gyrus of adult monkeys is diminished by stress. Proc. Natl. Acad. Sci. U S A 95, 3168–3171. 10.1073/pnas.95.6.3168 9501234 PMC19713

[B124] Grad L. I. Yerbury J. J. Turner B. J. Guest W. C. Pokrishevsky E. O’Neill M. A.. (2014). Intercellular propagated misfolding of wild-type Cu/Zn superoxide dismutase occurs via exosome-dependent and -independent mechanisms. Proc. Natl. Acad. Sci. U S A 111, 3620–3625. 10.1073/pnas.1312245111 24550511 PMC3948312

[B125] Graner M. W. Alzate O. Dechkovskaia A. M. Keene J. D. Sampson J. H. Mitchell D. A.. (2009). Proteomic and immunologic analyses of brain tumor exosomes. FASEB J. 23, 1541–1557. 10.1096/fj.08-122184 19109410 PMC2669426

[B126] Grapp M. Wrede A. Schweizer M. Hüwel S. Galla H. J. Snaidero N.. (2013). Choroid plexus transcytosis and exosome shuttling deliver folate into brain parenchyma. Nat. Commun. 4:2123. 10.1038/ncomms3123 23828504

[B127] Greening D. W. Gopal S. K. Mathias R. A. Liu L. Sheng J. Zhu H. J.. (2015). Emerging roles of exosomes during epithelial-mesenchymal transition and cancer progression. Semin. Cell Dev. Biol. 40, 60–71. 10.1016/j.semcdb.2015.02.008 25721809

[B128] Gregg C. Weiss S. (2005). CNTF/LIF/gp130 receptor complex signaling maintains a VZ precursor differentiation gradient in the developing ventral forebrain. Development 132, 565–578. 10.1242/dev.01592 15634701

[B129] Gross C. G. (2000). Neurogenesis in the adult brain: death of a dogma. Nat. Rev. Neurosci. 1, 67–73. 10.1038/35036235 11252770

[B130] Grote H. E. Hannan A. J. (2007). Regulators of adult neurogenesis in the healthy and diseased brain. Clin. Exp. Pharmacol. Physiol. 34, 533–545. 10.1111/j.1440-1681.2007.04610.x 17439429

[B131] Guescini M. Genedani S. Stocchi V. Agnati L. F. (2010). Astrocytes and Glioblastoma cells release exosomes carrying mtDNA. J. Neural Transm. (Vienna) 117, 1–4. 10.1007/s00702-009-0288-8 19680595

[B132] Gupta A. Pulliam L. (2014). Exosomes as mediators of neuroinflammation. J. Neuroinflammation 11:68. 10.1186/1742-2094-11-68 24694258 PMC3994210

[B133] György B. Hung M. E. Breakefield X. O. Leonard J. N. (2015). Therapeutic applications of extracellular vesicles: clinical promise and open questions. Annu. Rev. Pharmacol. Toxicol. 55, 439–464. 10.1146/annurev-pharmtox-010814-124630 25292428 PMC4445965

[B134] Hajrasouliha A. R. Jiang G. Lu Q. Lu H. Kaplan H. J. Zhang H. G.. (2013). Exosomes from retinal astrocytes contain antiangiogenic components that inhibit laser-induced choroidal neovascularization. J. Biol. Chem. 288, 28058–28067. 10.1074/jbc.M113.470765 23926109 PMC3784718

[B135] Hanson P. I. Cashikar A. (2012). Multivesicular body morphogenesis. Annu. Rev. Cell Dev. Biol. 28, 337–362. 10.1146/annurev-cellbio-092910-154152 22831642

[B136] Haqqani A. S. Delaney C. E. Tremblay T. L. Sodja C. Sandhu J. K. Stanimirovic D. B. (2013). Method for isolation and molecular characterization of extracellular microvesicles released from brain endothelial cells. Fluids Barriers CNS 10:4. 10.1186/2045-8118-10-4 23305214 PMC3560214

[B137] Harding C. Stahl P. (1983). Transferrin recycling in reticulocytes: pH and iron are important determinants of ligand binding and processing. Biochem. Biophys. Res. Commun. 113, 650–658. 10.1016/0006-291x(83)91776-x 6870878

[B138] Harry G. J. (2013). Microglia during development and aging. Pharmacol. Ther. 139, 313–326. 10.1016/j.pharmthera.2013.04.013 23644076 PMC3737416

[B139] He X. J. Nakayama H. (2015). Transiently impaired neurogenesis in MPTP mouse model of Parkinson’s disease. Neurotoxicology 50, 46–55. 10.1016/j.neuro.2015.07.007 26215120

[B140] Hellwig S. Heinrich A. Biber K. (2013). The brain’s best friend: microglial neurotoxicity revisited. Front. Cell. Neurosci. 7:71. 10.3389/fncel.2013.00071 23734099 PMC3655268

[B141] Hetz C. Mollereau B. (2014). Disturbance of endoplasmic reticulum proteostasis in neurodegenerative diseases. Nat. Rev. Neurosci. 15, 233–249. 10.1038/nrn3689 24619348

[B142] Higginbotham J. N. Demory Beckler M. Gephart J. D. Franklin J. L. Bogatcheva G. Kremers G. J.. (2011). Amphiregulin exosomes increase cancer cell invasion. Curr. Biol. 21, 779–786. 10.1016/j.cub.2011.03.043 21514161 PMC3417320

[B143] Höglinger G. U. Rizk P. Muriel M. P. Duyckaerts C. Oertel W. H. Caille I.. (2004). Dopamine depletion impairs precursor cell proliferation in Parkinson disease. Nat. Neurosci. 7, 726–735. 10.1038/nn1265 15195095

[B144] Hong C. S. Muller L. Whiteside T. L. Boyiadzis M. (2014). Plasma exosomes as markers of therapeutic response in patients with acute myeloid leukemia. Front. Immunol. 5:160. 10.3389/fimmu.2014.00160 24782865 PMC3989594

[B145] Hooper C. Sainz-Fuertes R. Lynham S. Hye A. Killick R. Warley A.. (2012). Wnt3a induces exosome secretion from primary cultured rat microglia. BMC Neurosci. 13:144. 10.1186/1471-2202-13-144 23173708 PMC3541220

[B146] Hosseini H. M. Fooladi A. A. Nourani M. R. Ghanezadeh F. (2013). The role of exosomes in infectious diseases. Inflamm. Allergy Drug Targets 12, 29–37. 10.2174/1871528111312010005 23441990

[B147] Hsu C. Morohashi Y. Yoshimura S. Manrique-Hoyos N. Jung S. Lauterbach M. A.. (2010). Regulation of exosome secretion by Rab35 and its GTPase-activating proteins TBC1D10A-C. J. Cell Biol. 189, 223–232. 10.1083/jcb.200911018 20404108 PMC2856897

[B148] Huang X. Yuan T. Tschannen M. Sun Z. Jacob H. Du M.. (2013). Characterization of human plasma-derived exosomal RNAs by deep sequencing. BMC Genomics 14:319. 10.1186/1471-2164-14-319 23663360 PMC3653748

[B149] Hurley J. H. Odorizzi G. (2012). Get on the exosome bus with ALIX. Nat. Cell Biol. 14, 654–655. 10.1038/ncb2530 22743708

[B150] Huttner H. B. Janich P. Köhrmann M. Jászai J. Siebzehnrubl F. Blümcke I.. (2008). The stem cell marker prominin-1/CD133 on membrane particles in human cerebrospinal fluid offers novel approaches for studying central nervous system disease. Stem Cells 26, 698–705. 10.1634/stemcells.2007-0639 18096722

[B151] Ibañez-Tallon I. Pagenstecher A. Fliegauf M. Olbrich H. Kispert A. Ketelsen U. P.. (2004). Dysfunction of axonemal dynein heavy chain Mdnah5 inhibits ependymal flow and reveals a novel mechanism for hydrocephalus formation. Hum. Mol. Genet. 13, 2133–2141. 10.1093/hmg/ddh219 15269178

[B152] Ioannidis J. P. Allison D. B. Ball C. A. Coulibaly I. Cui X. Culhane A. C.. (2009). Repeatability of published microarray gene expression analyses. Nat. Genet. 41, 149–155. 10.1038/ng.295 19174838

[B153] Ishitsuka K. Ago T. Arimura K. Nakamura K. Tokami H. Makihara N.. (2012). Neurotrophin production in brain pericytes during hypoxia: a role of pericytes for neuroprotection. Microvasc. Res. 83, 352–359. 10.1016/j.mvr.2012.02.009 22387236

[B154] Jacobs B. L. van Praag H. Gage F. H. (2000). Adult brain neurogenesis and psychiatry: a novel theory of depression. Mol. Psychiatry 5, 262–269. 10.1038/sj.mp.4000712 10889528

[B155] Jaiswal J. K. Andrews N. W. Simon S. M. (2002). Membrane proximal lysosomes are the major vesicles responsible for calcium-dependent exocytosis in nonsecretory cells. J. Cell Biol. 159, 625–635. 10.1083/jcb.200208154 12438417 PMC2173094

[B156] Jiménez A. J. Garcéa-Verdugo J. M. González C. A. Bátiz L. F. Rodríguez-Pérez L. M. Páez P.. (2009). Disruption of the neurogenic niche in the subventricular zone of postnatal hydrocephalic hyh mice. J. Neuropathol. Exp. Neurol. 68, 1006–1020. 10.1097/NEN.0b013e3181b44a5a 19680142

[B157] Jiménez A. J. Tomé M. Páez P. Wagner C. Rodréguez S. Fernández-Llebrez P.. (2001). A programmed ependymal denudation precedes congenital hydrocephalus in the hyh mutant mouse. J. Neuropathol. Exp. Neurol. 60, 1105–1119. 11706940 10.1093/jnen/60.11.1105

[B158] Jin K. Zhu Y. Sun Y. Mao X. O. Xie L. Greenberg D. A. (2002). Vascular endothelial growth factor (VEGF) stimulates neurogenesis *in vitro* and *in vivo*. Proc. Natl. Acad. Sci. U S A 99, 11946–11950. 10.1073/pnas.182296499 12181492 PMC129374

[B159] Johnstone R. M. Adam M. Hammond J. R. Orr L. Turbide C. (1987). Vesicle formation during reticulocyte maturation. Association of plasma membrane activities with released vesicles (exosomes). J. Biol. Chem. 262, 9412–9420. 3597417

[B160] Jordan J. D. Ma D. K. Ming G. L. Song H. (2007). Cellular niches for endogenous neural stem cells in the adult brain. CNS Neurol. Disord. Drug Targets 6, 336–341. 10.2174/187152707783220866 18045162

[B161] Jun H. Mohammed Qasim Hussaini S. Rigby M. J. Jang M. H. (2012). Functional role of adult hippocampal neurogenesis as a therapeutic strategy for mental disorders. Neural Plast. 2012:854285. 10.1155/2012/854285 23346419 PMC3549353

[B162] Jung K. H. Chu K. Lee S. T. Park H. K. Bahn J. J. Kim D. H.. (2009). Circulating endothelial microparticles as a marker of cerebrovascular disease. Ann. Neurol. 66, 191–199. 10.1002/ana.21681 19743467

[B163] Kajimoto T. Okada T. Miya S. Zhang L. Nakamura S. (2013). Ongoing activation of sphingosine 1-phosphate receptors mediates maturation of exosomal multivesicular endosomes. Nat. Commun. 4:2712. 10.1038/ncomms3712 24231649

[B164] Kalani A. Tyagi A. Tyagi N. (2014). Exosomes: mediators of neurodegeneration, neuroprotection and therapeutics. Mol. Neurobiol. 49, 590–600. 10.1007/s12035-013-8544-1 23999871 PMC3951279

[B165] Kang D. Oh S. Ahn S. M. Lee B. H. Moon M. H. (2008). Proteomic analysis of exosomes from human neural stem cells by flow field-flow fractionation and nanoflow liquid chromatography-tandem mass spectrometry. J. Proteome. Res. 7, 3475–3480. 10.1021/pr800225z 18570454

[B166] Kaplan M. S. (2001). Environment complexity stimulates visual cortex neurogenesis: death of a dogma and a research career. Trends Neurosci. 24, 617–620. 10.1016/s0166-2236(00)01967-6 11576677

[B167] Kapogiannis D. Boxer A. Schwartz J. B. Abner E. L. Biragyn A. Masharani U.. (2015). Dysfunctionally phosphorylated type 1 insulin receptor substrate in neural-derived blood exosomes of preclinical Alzheimer’s disease. FASEB J. 29, 589–596. 10.1096/fj.14-262048 25342129 PMC4314222

[B169] Katon W. J. (2008). The comorbidity of diabetes mellitus and depression. Am. J. Med. 121, S8–S15. 10.1016/j.amjmed.2008.09.008 18954592 PMC2717744

[B168] Katon W. Fan M. Y. Unützer J. Taylor J. Pincus H. Schoenbaum M. (2008). Depression and diabetes: a potentially lethal combination. J. Gen. Intern Med. 23, 1571–1575. 10.1007/s11606-008-0731-9 18649108 PMC2533367

[B170] Katsimpardi L. Litterman N. K. Schein P. A. Miller C. M. Loffredo F. S. Wojtkiewicz G. R.. (2014). Vascular and neurogenic rejuvenation of the aging mouse brain by young systemic factors. Science 344, 630–634. 10.1126/science.1251141 24797482 PMC4123747

[B171] Kawikova I. Askenase P. W. (2015). Diagnostic and therapeutic potentials of exosomes in CNS diseases. Brain Res. 1617, 63–71. 10.1016/j.brainres.2014.09.070 25304360 PMC4862949

[B172] Kempermann G. Song H. Gage F. H. (2015). Neurogenesis in the adult hippocampus. Cold Spring Harb Perspect. Med. 5:a018812. 26330519 10.1101/cshperspect.a018812 PMC4563705

[B173] Kernie S. G. Erwin T. M. Parada L. F. (2001). Brain remodeling due to neuronal and astrocytic proliferation after controlled cortical injury in mice. J. Neurosci. Res. 66, 317–326. 10.1002/jnr.10013 11746349

[B174] Kessler R. C. Ormel J. Demler O. Stang P. E. (2003). Comorbid mental disorders account for the role impairment of commonly occurring chronic physical disorders: results from the national comorbidity survey. J. Occup. Environ. Med. 45, 1257–1266. 10.1097/01.jom.0000100000.70011.bb 14665811

[B176] Kim S. U. de Vellis J. (2005). Microglia in health and disease. J. Neurosci. Res. 81, 302–313. 10.1002/jnr.20562 15954124

[B175] Kim H. Li Q. Hempstead B. L. Madri J. A. (2004). Paracrine and autocrine functions of brain-derived neurotrophic factor (BDNF) and nerve growth factor (NGF) in brain-derived endothelial cells. J. Biol. Chem. 279, 33538–33546. 10.1074/jbc.m404115200 15169782

[B177] Kippin T. E. Kapur S. van der Kooy D. (2005). Dopamine specifically inhibits forebrain neural stem cell proliferation, suggesting a novel effect of antipsychotic drugs. J. Neurosci. 25, 5815–5823. 10.1523/jneurosci.1120-05.2005 15958748 PMC6724880

[B178] Kirby E. D. Kuwahara A. A. Messer R. L. Wyss-Coray T. (2015). Adult hippocampal neural stem and progenitor cells regulate the neurogenic niche by secreting VEGF. Proc. Natl. Acad. Sci. U S A 112, 4128–4133. 10.1073/pnas.1422448112 25775598 PMC4386397

[B179] Klumperman J. Raposo G. (2014). The complex ultrastructure of the endolysosomal system. Cold Spring Harb. Perspect. Biol. 6:a016857. 10.1101/cshperspect.a016857 24851870 PMC4176003

[B180] Kokoeva M. V. Yin H. Flier J. S. (2005). Neurogenesis in the hypothalamus of adult mice: potential role in energy balance. Science 310, 679–683. 10.1126/science.1115360 16254185

[B181] Kokovay E. Goderie S. Wang Y. Lotz S. Lin G. Sun Y.. (2010). Adult SVZ lineage cells home to and leave the vascular niche via differential responses to SDF1/CXCR4 signaling. Cell Stem Cell 7, 163–173. 10.1016/j.stem.2010.05.019 20682445 PMC2916873

[B182] Kondabolu S. Adsumelli R. Schabel J. Glass P. Pentyala S. (2011). Evaluation of prostaglandin D2 as a CSF leak marker: implications in safe epidural anesthesia. Local Reg. Anesth. 4, 21–24. 10.2147/LRA.s18053 22915888 PMC3417968

[B183] Korkut C. Li Y. Koles K. Brewer C. Ashley J. Yoshihara M.. (2013). Regulation of postsynaptic retrograde signaling by presynaptic exosome release. Neuron 77, 1039–1046. 10.1016/j.neuron.2013.01.013 23522040 PMC3626103

[B184] Kowal J. Tkach M. Théry C. (2014). Biogenesis and secretion of exosomes. Curr. Opin. Cell Biol. 29, 116–125. 10.1016/j.ceb.2014.05.004 24959705

[B185] Krämer-Albers E. M. Bretz N. Tenzer S. Winterstein C. Möbius W. Berger H.. (2007). Oligodendrocytes secrete exosomes containing major myelin and stress-protective proteins: trophic support for axons? Proteomics Clin. Appl. 1, 1446–1461. 10.1002/prca.200700522 21136642

[B186] Kriegstein A. Alvarez-Buylla A. (2009). The glial nature of embryonic and adult neural stem cells. Annu. Rev. Neurosci. 32, 149–184. 10.1146/annurev.neuro.051508.135600 19555289 PMC3086722

[B187] Kuhlmann J. D. Hein L. Kurth I. Wimberger P. Dubrovska A. (2015). Targeting cancer stem cells: promises and challenges. Anticancer. Agents Med. Chem. 16, 38–58. 10.2174/1871520615666150716104152 26179271

[B188] Kunze A. Congreso M. R. Hartmann C. Wallraff-Beck A. Hüttmann K. Bedner P.. (2009). Connexin expression by radial glia-like cells is required for neurogenesis in the adult dentate gyrus. Proc. Natl. Acad. Sci. U S A 106, 11336–11341. 10.1073/pnas.0813160106 19549869 PMC2700144

[B189] Lachenal G. Pernet-Gallay K. Chivet M. Hemming F. J. Belly A. Bodon G.. (2011). Release of exosomes from differentiated neurons and its regulation by synaptic glutamatergic activity. Mol. Cell. Neurosci. 46, 409–418. 10.1016/j.mcn.2010.11.004 21111824

[B190] Lafon-Cazal M. Adjali O. Galeotti N. Poncet J. Jouin P. Homburger V.. (2003). Proteomic analysis of astrocytic secretion in the mouse. Comparison with the cerebrospinal fluid proteome. J. Biol. Chem. 278, 24438–24448. 10.1074/jbc.m211980200 12709418

[B191] Lai R. C. Chen T. S. Lim S. K. (2011). Mesenchymal stem cell exosome: a novel stem cell-based therapy for cardiovascular disease. Regen. Med. 6, 481–492. 10.2217/rme.11.35 21749206

[B192] Lai R. C. Yeo R. W. Lim S. K. (2015). Mesenchymal stem cell exosomes. Semin Cell Dev. Biol. 40, 82–88. 10.1016/j.semcdb.2015.03.001-5 25765629

[B193] Lange S. Trost A. Tempfer H. Bauer H. C. Bauer H. Rohde E.. (2013). Brain pericyte plasticity as a potential drug target in CNS repair. Drug Discov. Today 18, 456–463. 10.1016/j.drudis.2012.12.007 23266366

[B194] Le Grand J. N. Gonzalez-Cano L. Pavlou M. A. Schwamborn J. C. (2015). Neural stem cells in Parkinson’s disease: a role for neurogenesis defects in onset and progression. Cell Mol. Life Sci. 72, 773–797. 10.1007/s00018-014-1774-1 25403878 PMC11113294

[B195] Leranth C. Hajszan T. (2007). Extrinsic afferent systems to the dentate gyrus. Prog. Brain Res. 163, 63–84. 10.1016/s0079-6123(07)63004-0 17765712 PMC1989689

[B196] Li L. Xie T. (2005). Stem cell niche: structure and function. Annu. Rev. Cell Dev. Biol. 21, 605–631. 10.1146/annurev.cellbio.21.012704.131525 16212509

[B197] Licht T. Keshet E. (2015). The vascular niche in adult neurogenesis. Mech. Dev. 138, 56–62. 10.1016/j.mod.2015.06.001 26103548

[B198] Liddelow S. A. Temple S. Møllgård K. Gehwolf R. Wagner A. Bauer H.. (2012). Molecular characterisation of transport mechanisms at the developing mouse blood-CSF interface: a transcriptome approach. PLoS One 7:e33554. 10.1371/journal.pone.0033554 22457777 PMC3310074

[B199] Lie D. C. Colamarino S. A. Song H. J. Désiré L. Mira H. Consiglio A.. (2005). Wnt signalling regulates adult hippocampal neurogenesis. Nature 437, 1370–1375. 10.1038/nature04108 16251967

[B200] Lie D. C. Song H. Colamarino S. A. Ming G. L. Gage F. H. (2004). Neurogenesis in the adult brain: new strategies for central nervous system diseases. Annu. Rev. Pharmacol. Toxicol. 44, 399–421. 10.1007/1-4020-2541-6_26 14744252

[B201] Lim D. A. Tramontin A. D. Trevejo J. M. Herrera D. G. Garcia-Verdugo J. M. Alvarez-Buylla A. (2000). Noggin antagonizes BMP signaling to create a niche for adult neurogenesis. Neuron 28, 713–726. 10.1016/s0896-6273(00)00148-3 11163261

[B202] Lin R. Iacovitti L. (2015). Classic and novel stem cell niches in brain homeostasis and repair. Brain Res. 1628, 327–342. 10.1016/j.brainres.2015.04.029 25931262

[B204] Liu X. S. Chopp M. Zhang R. L. Tao T. Wang X. L. Kassis H.. (2011). MicroRNA profiling in subventricular zone after stroke: MiR-124a regulates proliferation of neural progenitor cells through Notch signaling pathway. PLoS One 6:e23461. 10.1371/journal.pone.0023461 21887253 PMC3162555

[B203] Liu X. Wang Q. Haydar T. F. Bordey A. (2005). Nonsynaptic GABA signaling in postnatal subventricular zone controls proliferation of GFAP-expressing progenitors. Nat. Neurosci. 8, 1179–1187. 10.1038/nn1522 16116450 PMC1380263

[B205] Lledo P. M. Alonso M. Grubb M. S. (2006). Adult neurogenesis and functional plasticity in neuronal circuits. Nat. Rev. Neurosci. 7, 179–193. 10.1038/nrn1867 16495940

[B206] Lopez-Verrilli M. A. Court F. A. (2013). Exosomes: mediators of communication in eukaryotes. Biol Res 46, 5–11. 10.4067/s0716-97602013000100001 23760408

[B207] Low L. H. Chow Y. L. Li Y. Goh C. P. Putz U. Silke J.. (2015). Nedd4 family interacting protein 1 (Ndfip1) is required for ubiquitination and nuclear trafficking of BRCA1-associated ATM activator 1 (BRAT1) during the DNA damage response. J. Biol. Chem. 290, 7141–7150. 10.1074/jbc.m114.613687 25631046 PMC4358134

[B208] Lu Z. Kipnis J. (2010). Thrombospondin 1—a key astrocyte-derived neurogenic factor. FASEB J. 24, 1925–1934. 10.1096/fj.09-150573 20124433 PMC3231793

[B209] Lugert S. Basak O. Knuckles P. Haussler U. Fabel K. Götz M.. (2010). Quiescent and active hippocampal neural stem cells with distinct morphologies respond selectively to physiological and pathological stimuli and aging. Cell Stem Cell 6, 445–456. 10.1016/j.stem.2010.03.017 20452319

[B212] Luo X. G. Chen S. D. (2012). The changing phenotype of microglia from homeostasis to disease. Transl. Neurodegener. 1:9. 10.1186/2047-9158-1-9 23210447 PMC3514090

[B211] Luo L. Craik F. I. (2008). Aging and memory: a cognitive approach. Can. J. Psychiatry 53, 346–353. 18616854 10.1177/070674370805300603

[B210] Luo J. Shook B. A. Daniels S. B. Conover J. C. (2008). Subventricular zone-mediated ependyma repair in the adult mammalian brain. J. Neurosci. 28, 3804–3813. 10.1523/JNEUROSCI.0224-08.2008 18385338 PMC2638095

[B214] Ma D. K. Ming G. L. Gage F. H. Song H. (2008). “Neurogenic niches in the adult mammalian brain,” in Adult Neurogenesis, eds Gage F. H. Kempermann G. Song H. (New York, NY: Cold Spring Harbor Laboratory Press), 207–225.

[B213] Ma D. K. Ming G. L. Song H. (2005). Glial influences on neural stem cell development: cellular niches for adult neurogenesis. Curr. Opin. Neurobiol. 15, 514–520. 10.1016/j.conb.2005.08.003 16144763

[B215] Ma D. K. Ponnusamy K. Song M. R. Ming G. L. Song H. (2009). Molecular genetic analysis of FGFR1 signalling reveals distinct roles of MAPK and PLCgamma1 activation for self-renewal of adult neural stem cells. Mol. Brain 2:16. 10.1186/1756-6606-2-16 19505325 PMC2700800

[B216] Malberg J. E. Eisch A. J. Nestler E. J. Duman R. S. (2000). Chronic antidepressant treatment increases neurogenesis in adult rat hippocampus. J. Neurosci. 20, 9104–9110. 11124987 10.1523/JNEUROSCI.20-24-09104.2000 PMC6773038

[B217] Markwardt S. J. Wadiche J. I. Overstreet-Wadiche L. S. (2009). Input-specific GABAergic signaling to newborn neurons in adult dentate gyrus. J. Neurosci. 29, 15063–15072. 10.1523/JNEUROSCI.2727-09.2009 19955357 PMC2846629

[B218] Marques F. Sousa J. C. Coppola G. Gao F. Puga R. Brentani H.. (2011). Transcriptome signature of the adult mouse choroid plexus. Fluids Barriers CNS 8:10. 10.1186/2045-8118-8-10 21349147 PMC3042978

[B219] Marzesco A. M. Janich P. Wilsch-Bräuninger M. Dubreuil V. Langenfeld K. Corbeil D.. (2005). Release of extracellular membrane particles carrying the stem cell marker prominin-1 (CD133) from neural progenitors and other epithelial cells. J. Cell Sci. 118, 2849–2858. 10.1242/jcs.02439 15976444

[B220] Mashayekhi F. Draper C. E. Bannister C. M. Pourghasem M. Owen-Lynch P. J. Miyan J. A. (2002). Deficient cortical development in the hydrocephalic Texas (H-Tx) rat: a role for CSF. Brain 125, 1859–1874. 10.1093/brain/awf182 12135976

[B221] Mathivanan S. Fahner C. J. Reid G. E. Simpson R. J. (2012). ExoCarta 2012: database of exosomal proteins, RNA and lipids. Nucleic Acids Res. 40, D1241–D1244. 10.1093/nar/gkr828 21989406 PMC3245025

[B222] Mathivanan S. Lim J. W. Tauro B. J. Ji H. Moritz R. L. Simpson R. J. (2010). Proteomics analysis of A33 immunoaffinity-purified exosomes released from the human colon tumor cell line LIM1215 reveals a tissue-specific protein signature. Mol. Cell. Proteomics 9, 197–208. 10.1074/mcp.M900152-MCP200 19837982 PMC2830834

[B223] Matsuo H. Chevallier J. Mayran N. Le Blanc I. Ferguson C. Fauré J.. (2004). Role of LBPA and Alix in multivesicular liposome formation and endosome organization. Science 303, 531–534. 10.1126/science.1092425 14739459

[B224] Menn B. Garcia-Verdugo J. M. Yaschine C. Gonzalez-Perez O. Rowitch D. Alvarez-Buylla A. (2006). Origin of oligodendrocytes in the subventricular zone of the adult brain. J. Neurosci. 26, 7907–7918. 10.1523/jneurosci.1299-06.2006 16870736 PMC6674207

[B225] Merkle F. T. Alvarez-Buylla A. (2006). Neural stem cells in mammalian development. Curr. Opin. Cell Biol. 18, 704–709. 10.1016/j.ceb.2006.09.008 17046226

[B226] Ming G. L. Song H. (2005). Adult neurogenesis in the mammalian central nervous system. Annu. Rev. Neurosci. 28, 223–250. 10.1146/annurev.neuro.28.051804.101459 16022595

[B227] Mirzadeh Z. Merkle F. T. Soriano-Navarro M. Garcia-Verdugo J. M. Alvarez-Buylla A. (2008). Neural stem cells confer unique pinwheel architecture to the ventricular surface in neurogenic regions of the adult brain. Cell Stem Cell 3, 265–278. 10.1016/j.stem.2008.07.004 18786414 PMC2613692

[B228] Mittelbrunn M. Sánchez-Madrid F. (2012). Intercellular communication: diverse structures for exchange of genetic information. Nat. Rev. Mol. Cell Biol. 13, 328–335. 10.1038/nrm3335 22510790 PMC3738855

[B229] Miyan J. A. Nabiyouni M. Zendah M. (2003). Development of the brain: a vital role for cerebrospinal fluid. Can. J. Physiol. Pharmacol. 81, 317–328. 10.1139/y03-027 12769224

[B230] Mollinari C. Racaniello M. Berry A. Pieri M. de Stefano M. C. Cardinale A.. (2015). miR-34a regulates cell proliferation, morphology and function of newborn neurons resulting in improved behavioural outcomes. Cell Death Dis. 6:e1622. 10.1038/cddis.2014.589 25633291 PMC4669781

[B231] Monje M. L. Toda H. Palmer T. D. (2003). Inflammatory blockade restores adult hippocampal neurogenesis. Science 302, 1760–1765. 10.1126/science.1088417 14615545

[B232] Morel L. Regan M. Higashimori H. Ng S. K. Esau C. Vidensky S.. (2013). Neuronal exosomal miRNA-dependent translational regulation of astroglial glutamate transporter GLT1. J. Biol. Chem. 288, 7105–7116. 10.1074/jbc.M112.410944 23364798 PMC3591620

[B233] Mu Y. Gage F. H. (2011). Adult hippocampal neurogenesis and its role in Alzheimer’s disease. Mol. Neurodegener. 6:85. 10.1186/1750-1326-6-85 22192775 PMC3261815

[B234] Mulcahy L. A. Pink R. C. Carter D. R. (2014). Routes and mechanisms of extracellular vesicle uptake. J. Extracell. Vesicles 3:24641. 10.3402/jev.v3.24641 25143819 PMC4122821

[B235] Muller L. Muller-Haegele S. Mitsuhashi M. Gooding W. Okada H. Whiteside T. L. (2015). Exosomes isolated from plasma of glioma patients enrolled in a vaccination trial reflect antitumor immune activity and might predict survival. Oncoimmunology 4:e1008347. 10.1080/2162402x.2015.1008347 26155415 PMC4485717

[B236] Nave K. A. Trapp B. D. (2008). Axon-glial signaling and the glial support of axon function. Annu. Rev. Neurosci. 31, 535–561. 10.1146/annurev.neuro.30.051606.094309 18558866

[B237] Nazarenko I. Rana S. Baumann A. McAlear J. Hellwig A. Trendelenburg M.. (2010). Cell surface tetraspanin Tspan8 contributes to molecular pathways of exosome-induced endothelial cell activation. Cancer Res. 70, 1668–1678. 10.1158/0008-5472.CAN-09-2470 20124479

[B238] Nelson D. J. Wright E. M. (1974). The distribution, activity and function of the cilia in the frog brain. J. Physiol. 243, 63–78. 10.1113/jphysiol.1974.sp010742 4375184 PMC1330689

[B239] Nguyen-Ba-Charvet K. T. Picard-Riera N. Tessier-Lavigne M. Baron-Van Evercooren A. Sotelo C. Chédotal A. (2004). Multiple roles for slits in the control of cell migration in the rostral migratory stream. J. Neurosci. 24, 1497–1506. 10.1523/jneurosci.4729-03.2004 14960623 PMC6730320

[B240] Nimmerjahn A. Kirchhoff F. Helmchen F. (2005). Resting microglial cells are highly dynamic surveillants of brain parenchyma *in vivo*. Science 308, 1314–1318. 10.1126/science.1110647 15831717

[B241] Njie E. G. Boelen E. Stassen F. R. Steinbusch H. W. Borchelt D. R. Streit W. J. (2012). Ex vivo cultures of microglia from young and aged rodent brain reveal age-related changes in microglial function. Neurobiol. Aging 33, 195.e1–195.e12. 10.1016/j.neurobiolaging.2010.05.008 20580465 PMC4162517

[B242] Oh I. H. (2010). Microenvironmental targeting of Wnt/beta-catenin signals for hematopoietic stem cell regulation. Expert Opin. Biol. Ther. 10, 1315–1329. 10.1517/14712598.2010.504705 20636187

[B243] Olah M. Ping G. De Haas A. H. Brouwer N. Meerlo P. Van Der Zee E. A.. (2009). Enhanced hippocampal neurogenesis in the absence of microglia T cell interaction and microglia activation in the murine running wheel model. Glia 57, 1046–1061. 10.1002/glia.20828 19115394

[B244] Oliver C. González C. A. Alvial G. Flores C. A. Rodríguez E. M. Bátiz L. F. (2013). Disruption of CDH2/N-cadherin-based adherens junctions leads to apoptosis of ependymal cells and denudation of brain ventricular walls. J. Neuropathol. Exp. Neurol. 72, 846–860. 10.1097/NEN.0b013e3182a2d5fe 23965744

[B245] Owen-Lynch P. J. Draper C. E. Mashayekhi F. Bannister C. M. Miyan J. A. (2003). Defective cell cycle control underlies abnormal cortical development in the hydrocephalic Texas rat. Brain 126, 623–631. 10.1093/brain/awg058 12566283

[B246] Páez P. Bátiz L. F. Roales-Buján R. Rodríguez-Pérez L. M. Rodríguez S. Jiménez A. J.. (2007). Patterned neuropathologic events occurring in hyh congenital hydrocephalic mutant mice. J. Neuropathol. Exp. Neurol. 66, 1082–1092. 10.1097/nen.0b013e31815c1952 18090917

[B247] Paez-Gonzalez P. Asrican B. Rodriguez E. Kuo C. T. (2014). Identification of distinct ChAT(+) neurons and activity-dependent control of postnatal SVZ neurogenesis. Nat. Neurosci. 17, 934–942. 10.1038/nn.3734 24880216 PMC4122286

[B248] Palmer T. D. Willhoite A. R. Gage F. H. (2000). Vascular niche for adult hippocampal neurogenesis. J. Comp. Neurol. 425, 479–494. 10.1002/1096-9861(20001002)425:4<479::aid-cne2>3.0.co;2-3 10975875

[B249] Pan B. T. Johnstone R. M. (1983). Fate of the transferrin receptor during maturation of sheep reticulocytes *in vitro*: selective externalization of the receptor. Cell 33, 967–978. 10.1016/0092-8674(83)90040-5 6307529

[B250] Parolini I. Federici C. Raggi C. Lugini L. Palleschi S. De Milito A.. (2009). Microenvironmental pH is a key factor for exosome traffic in tumor cells. J. Biol. Chem. 284, 34211–34222. 10.1074/jbc.M109.041152 19801663 PMC2797191

[B251] Paschon V. Takada S. H. Ikebara J. M. Sousa E. Raeisossadati R. Ulrich H.. (2015). Interplay between exosomes, micrornas and toll-like receptors in brain disorders. Mol. Neurobiol. [Epub ahead of print]. 10.1007/s12035-015-9142-1 25862375

[B252] Peferoen L. Kipp M. van der Valk P. van Noort J. M. Amor S. (2014). Oligodendrocyte-microglia cross-talk in the central nervous system. Immunology 141, 302–313. 10.1111/imm.12163 23981039 PMC3930369

[B253] Pegtel D. M. Peferoen L. Amor S. (2014). Extracellular vesicles as modulators of cell-to-cell communication in the healthy and diseased brain. Philos. Trans. R. Soc. Lond. B Biol. Sci. 369:20130516. 10.1098/rstb.2013.0516 25135977 PMC4142037

[B254] Peinado H. Alečković M. Lavotshkin S. Matei I. Costa-Silva B. Moreno-Bueno G.. (2012). Melanoma exosomes educate bone marrow progenitor cells toward a pro-metastatic phenotype through MET. Nat. Med. 18, 883–891. 10.1038/nm.2753 22635005 PMC3645291

[B255] Pitt J. M. Charrier M. Viaud S. André F. Besse B. Chaput N.. (2014). Dendritic cell-derived exosomes as immunotherapies in the fight against cancer. J. Immunol. 193, 1006–1011. 10.4049/jimmunol.1400703 25049431

[B256] Plaks V. Kong N. Werb Z. (2015). The cancer stem cell niche: how essential is the niche in regulating stemness of tumor cells? Cell Stem Cell 16, 225–238. 10.1016/j.stem.2015.02.015 25748930 PMC4355577

[B257] Pogue A. I. Cui J. G. Li Y. Y. Zhao Y. Culicchia F. Lukiw W. J. (2010). Micro RNA-125b (miRNA-125b) function in astrogliosis and glial cell proliferation. Neurosci. Lett. 476, 18–22. 10.1016/j.neulet.2010.03.054 20347935

[B258] Potolicchio I. Carven G. J. Xu X. Stipp C. Riese R. J. Stern L. J.. (2005). Proteomic analysis of microglia-derived exosomes: metabolic role of the aminopeptidase CD13 in neuropeptide catabolism. J. Immunol. 175, 2237–2243. 10.4049/jimmunol.175.4.2237 16081791

[B259] Prada I. Furlan R. Matteoli M. Verderio C. (2013). Classical and unconventional pathways of vesicular release in microglia. Glia 61, 1003–1017. 10.1002/glia.22497 23625857

[B260] Prado N. Marazuela E. G. Segura E. Fernández-García H. Villalba M. Théry C.. (2008). Exosomes from bronchoalveolar fluid of tolerized mice prevent allergic reaction. J. Immunol. 181, 1519–1525. 10.4049/jimmunol.181.2.1519 18606707

[B261] Proia P. Schiera G. Mineo M. Ingrassia A. M. Santoro G. Savettieri G.. (2008). Astrocytes shed extracellular vesicles that contain fibroblast growth factor-2 and vascular endothelial growth factor. Int. J. Mol. Med. 21, 63–67. 10.3892/ijmm.21.1.63 18097617

[B262] Properzi F. Logozzi M. Abdel-Haq H. Federici C. Lugini L. Azzarito T.. (2015). Detection of exosomal prions in blood by immunochemistry techniques. J. Gen. Virol. 96, 1969–1974. 10.1099/vir.0.000117 25805411

[B263] Putz U. Howitt J. Lackovic J. Foot N. Kumar S. Silke J.. (2008). Nedd4 family-interacting protein 1 (Ndfip1) is required for the exosomal secretion of Nedd4 family proteins. J. Biol. Chem. 283, 32621–32627. 10.1074/jbc.M804120200 18819914

[B264] Raimondo S. Saieva L. Corrado C. Fontana S. Flugy A. Rizzo A.. (2015). Chronic myeloid leukemia-derived exosomes promote tumor growth through an autocrine mechanism. Cell Commun. Signal. 13:8. 10.1186/s12964-015-0086-x 25644060 PMC4320527

[B265] Rajendran L. Honsho M. Zahn T. R. Keller P. Geiger K. D. Verkade P.. (2006). Alzheimer’s disease beta-amyloid peptides are released in association with exosomes. Proc. Natl. Acad. Sci. U S A 103, 11172–11177. 10.1073/pnas.0603838103 16837572 PMC1544060

[B266] Ramírez-Castillejo C. Sánchez-Sánchez F. Andreu-Agulló C. Ferrón S. R. Aroca-Aguilar J. D. Sánchez P.. (2006). Pigment epithelium-derived factor is a niche signal for neural stem cell renewal. Nat. Neurosci. 9, 331–339. 10.1038/nn1657 16491078

[B267] Raposo G. Nijman H. W. Stoorvogel W. Liejendekker R. Harding C. V. Melief C. J.. (1996). B lymphocytes secrete antigen-presenting vesicles. J. Exp. Med. 183, 1161–1172. 10.1084/jem.183.3.1161 8642258 PMC2192324

[B268] Redzic Z. B. Preston J. E. Duncan J. A. Chodobski A. Szmydynger-Chodobska J. (2005). The choroid plexus-cerebrospinal fluid system: from development to aging. Curr. Top. Dev. Biol. 71, 1–52. 10.1016/s0070-2153(05)71001-2 16344101

[B269] Regehr W. G. Carey M. R. Best A. R. (2009). Activity-dependent regulation of synapses by retrograde messengers. Neuron 63, 154–170. 10.1016/j.neuron.2009.06.021 19640475 PMC3251517

[B270] Regensburger M. Prots I. Winner B. (2014). Adult hippocampal neurogenesis in Parkinson’s disease: impact on neuronal survival and plasticity. Neural Plast. 2014:454696. 10.1155/2014/454696 25110593 PMC4106176

[B271] Ridder K. Keller S. Dams M. Rupp A. K. Schlaudraff J. Del Turco D.. (2014). Extracellular vesicle-mediated transfer of genetic information between the hematopoietic system and the brain in response to inflammation. PLoS Biol. 12:e1001874. 10.1371/journal.pbio.1001874 24893313 PMC4043485

[B272] Riquelme P. A. Drapeau E. Doetsch F. (2008). Brain micro-ecologies: neural stem cell niches in the adult mammalian brain. Philos. Trans. R. Soc. Lond. B Biol. Sci. 363, 123–137. 10.1098/rstb.2006.2016 17322003 PMC2605490

[B273] Roales-Buján R. Páez P. Guerra M. Rodríguez S. Vio K. Ho-Plagaro A.. (2012). Astrocytes acquire morphological and functional characteristics of ependymal cells following disruption of ependyma in hydrocephalus. Acta Neuropathol. 124, 531–546. 10.1007/s00401-012-0992-6 22576081 PMC3444707

[B274] Rodríguez P. Bouchaud C. (1996). The supra-ependymal innervation is not responsible for the repression of tight junctions in the rat cerebral ependyma. Neurobiology (Bp) 4, 185–201. 9044345

[B275] Ruiz de Almodovar C. Lambrechts D. Mazzone M. Carmeliet P. (2009). Role and therapeutic potential of VEGF in the nervous system. Physiol. Rev. 89, 607–648. 10.1152/physrev.00031.2008 19342615

[B276] Ryan B. Joilin G. Williams J. M. (2015). Plasticity-related microRNA and their potential contribution to the maintenance of long-term potentiation. Front. Mol. Neurosci. 8:4. 10.3389/fnmol.2015.00004 25755632 PMC4337328

[B277] Sáenz-Cuesta M. Osorio-Querejeta I. Otaegui D. (2014). Extracellular vesicles in multiple sclerosis: what are they telling us? Front. Cell. Neurosci. 8:100. 10.3389/fncel.2014.00100 24734004 PMC3975116

[B278] Saman S. Kim W. Raya M. Visnick Y. Miro S. Saman S.. (2012). Exosome-associated tau is secreted in tauopathy models and is selectively phosphorylated in cerebrospinal fluid in early Alzheimer disease. J. Biol. Chem. 287, 3842–3849. 10.1074/jbc.M111.277061 22057275 PMC3281682

[B279] Sandoval M. Luarte A. Herrera-Molina R. Varas-Godoy M. Santibáñez M. Rubio F. J.. (2013). The glycolytic enzyme aldolase C is up-regulated in rat forebrain microsomes and in the cerebrospinal fluid after repetitive fluoxetine treatment. Brain Res. 1520, 1–14. 10.1016/j.brainres.2013.04.049 23688545

[B280] Santarelli L. Saxe M. Gross C. Surget A. Battaglia F. Dulawa S.. (2003). Requirement of hippocampal neurogenesis for the behavioral effects of antidepressants. Science 301, 805–809. 10.1126/science.1083328 12907793

[B281] Santos M. C. Tegge A. N. Correa B. R. Mahesula S. Kohnke L. Q. Qiao M.. (2015). miR-124, -128 and -137 orchestrate neural differentiation by acting on overlapping gene sets containing a highly connected transcription factor network. Stem Cells [Epub ahead of print]. 10.1002/stem.2204 26369286

[B282] Sarnat H. B. (1992). Role of human fetal ependyma. Pediatr. Neurol. 8, 163–178. 10.1016/0887-8994(92)90063-5 1622511

[B283] Sato K. Malchinkhuu E. Horiuchi Y. Mogi C. Tomura H. Tosaka M.. (2007). HDL-like lipoproteins in cerebrospinal fluid affect neural cell activity through lipoprotein-associated sphingosine 1-phosphate. Biochem. Biophys. Res. Commun. 359, 649–654. 10.1016/j.bbrc.2007.05.131 17544365

[B284] Sawamoto K. Wichterle H. Gonzalez-Perez O. Cholfin J. A. Yamada M. Spassky N.. (2006). New neurons follow the flow of cerebrospinal fluid in the adult brain. Science 311, 629–632. 10.1126/science.1119133 16410488

[B285] Scadden D. T. (2006). The stem-cell niche as an entity of action. Nature 441, 1075–1079. 10.1038/nature04957 16810242

[B286] Schofield R. (1978). The relationship between the spleen colony-forming cell and the haemopoietic stem cell. Blood Cells 4, 7–25. 747780

[B287] Seri B. García-Verdugo J. M. McEwen B. S. Alvarez-Buylla A. (2001). Astrocytes give rise to new neurons in the adult mammalian hippocampus. J. Neurosci. 21, 7153–7160. 11549726 10.1523/JNEUROSCI.21-18-07153.2001 PMC6762987

[B288] Sette P. Jadwin J. A. Dussupt V. Bello N. F. Bouamr F. (2010). The ESCRT-associated protein Alix recruits the ubiquitin ligase Nedd4–1 to facilitate HIV-1 release through the LYPXnL L domain motif. J. Virol. 84, 8181–8192. 10.1128/JVI.00634-10 20519395 PMC2916511

[B289] Shao H. Chung J. Balaj L. Charest A. Bigner D. D. Carter B. S.. (2012). Protein typing of circulating microvesicles allows real-time monitoring of glioblastoma therapy. Nat. Med. 18, 1835–1840. 10.1038/nm.2994 23142818 PMC3518564

[B290] Shapiro L. A. Korn M. J. Shan Z. Ribak C. E. (2005). GFAP-expressing radial glia-like cell bodies are involved in a one-to-one relationship with doublecortin-immunolabeled newborn neurons in the adult dentate gyrus. Brain Res. 1040, 81–91. 10.1016/j.brainres.2005.01.098 15804429

[B291] Sharghi-Namini S. Tan E. Ong L. L. Ge R. Asada H. H. (2014). Dll4-containing exosomes induce capillary sprout retraction in a 3D microenvironment. Sci. Rep. 4:4031. 10.1038/srep04031 24504253 PMC3916896

[B292] Sheldon H. Heikamp E. Turley H. Dragovic R. Thomas P. Oon C. E.. (2010). New mechanism for Notch signaling to endothelium at a distance by Delta-like 4 incorporation into exosomes. Blood 116, 2385–2394. 10.1182/blood-2009-08-239228 20558614

[B293] Shen Q. Goderie S. K. Jin L. Karanth N. Sun Y. Abramova N.. (2004). Endothelial cells stimulate self-renewal and expand neurogenesis of neural stem cells. Science 304, 1338–1340. 10.1126/science.1095505 15060285

[B294] Shen Q. Wang Y. Kokovay E. Lin G. Chuang S. M. Goderie S. K.. (2008). Adult SVZ stem cells lie in a vascular niche: a quantitative analysis of niche cell-cell interactions. Cell Stem Cell 3, 289–300. 10.1016/j.stem.2008.07.026 18786416 PMC2747473

[B295] Shetty A. K. Hattiangady B. Shetty G. A. (2005). Stem/progenitor cell proliferation factors FGF-2, IGF-1 and VEGF exhibit early decline during the course of aging in the hippocampus: role of astrocytes. Glia 51, 173–186. 10.1002/glia.20187 15800930

[B296] Shi M. Liu C. Cook T. J. Bullock K. M. Zhao Y. Ginghina C.. (2014). Plasma exosomal alpha-synuclein is likely CNS-derived and increased in Parkinson’s disease. Acta Neuropathol. 128, 639–650. 10.1007/s00401-014-1314-y 24997849 PMC4201967

[B297] Shields R. (2006). MIAME, we have a problem. Trends Genet. 22, 65–66. 10.1016/j.tig.2005.12.006 16380192

[B298] Shimazaki T. Shingo T. Weiss S. (2001). The ciliary neurotrophic factor/leukemia inhibitory factor/gp130 receptor complex operates in the maintenance of mammalian forebrain neural stem cells. J. Neurosci. 21, 7642–7653. 11567054 10.1523/JNEUROSCI.21-19-07642.2001 PMC6762896

[B299] Shingo T. Gregg C. Enwere E. Fujikawa H. Hassam R. Geary C.. (2003). Pregnancy-stimulated neurogenesis in the adult female forebrain mediated by prolactin. Science 299, 117–120. 10.1126/science.1076647 12511652

[B300] Shruster A. Melamed E. Offen D. (2010). Neurogenesis in the aged and neurodegenerative brain. Apoptosis 15, 1415–1421. 10.1007/s10495-010-0491-y 20339917

[B301] Sierra A. Abiega O. Shahraz A. Neumann H. (2013). Janus-faced microglia: beneficial and detrimental consequences of microglial phagocytosis. Front. Cell. Neurosci. 7:6. 10.3389/fncel.2013.00006 23386811 PMC3558702

[B302] Sierra A. Beccari S. Diaz-Aparicio I. Encinas J. M. Comeau S. Tremblay M. E. (2014). Surveillance, phagocytosis and inflammation: how never-resting microglia influence adult hippocampal neurogenesis. Neural Plast. 2014:610343. 10.1155/2014/610343 24772353 PMC3977558

[B303] Sierra A. Encinas J. M. Deudero J. J. Chancey J. H. Enikolopov G. Overstreet-Wadiche L. S.. (2010). Microglia shape adult hippocampal neurogenesis through apoptosis-coupled phagocytosis. Cell Stem Cell 7, 483–495. 10.1016/j.stem.2010.08.014 20887954 PMC4008496

[B304] Simak J. Gelderman M. P. Yu H. Wright V. Baird A. E. (2006). Circulating endothelial microparticles in acute ischemic stroke: a link to severity, lesion volume and outcome. J. Thromb. Haemost. 4, 1296–1302. 10.1111/j.1538-7836.2006.01911.x 16706974

[B305] Sims B. Gu L. Krendelchtchikov A. Matthews Q. L. (2014). Neural stem cell-derived exosomes mediate viral entry. Int. J. Nanomedicine 9, 4893–4897. 10.2147/IJN.S70999 25364247 PMC4211904

[B306] Sival D. A. Guerra M. den Dunnen W. F. Bátiz L. F. Alvial G. Castañeyra-Perdomo A.. (2011). Neuroependymal denudation is in progress in full-term human foetal spina bifida aperta. Brain Pathol. 21, 163–179. 10.1111/j.1750-3639.2010.00432.x 21269337 PMC8094240

[B307] Skog J. Würdinger T. van Rijn S. Meijer D. H. Gainche L. Sena-Esteves M.. (2008). Glioblastoma microvesicles transport RNA and proteins that promote tumour growth and provide diagnostic biomarkers. Nat. Cell Biol. 10, 1470–1476. 10.1038/ncb1800 19011622 PMC3423894

[B308] Smalheiser N. R. (2007). Exosomal transfer of proteins and RNAs at synapses in the nervous system. Biol. Direct 2:35. 10.1186/1745-6150-2-35 18053135 PMC2219957

[B309] Smythies J. Edelstein L. (2013). Transsynaptic modality codes in the brain: possible involvement of synchronized spike timing, microRNAs, exosomes and epigenetic processes. Front. Integr. Neurosci. 6:126. 10.3389/fnint.2012.00126 23316146 PMC3539687

[B310] Snyder J. S. Soumier A. Brewer M. Pickel J. Cameron H. A. (2011). Adult hippocampal neurogenesis buffers stress responses and depressive behaviour. Nature 476, 458–461. 10.1038/nature10287 21814201 PMC3162077

[B311] Soares A. R. Martins-Marques T. Ribeiro-Rodrigues T. Ferreira J. V. Catarino S. Pinho M. J.. (2015). Gap junctional protein Cx43 is involved in the communication between extracellular vesicles and mammalian cells. Sci. Rep. 5:13243. 10.1038/srep13243 26285688 PMC4541155

[B312] Solé C. Cortés-Hernández J. Felip M. L. Vidal M. Ordi-Ros J. (2015). miR-29c in urinary exosomes as predictor of early renal fibrosis in lupus nephritis. Nephrol. Dial. Transplant. 30, 1488–1496. 10.1093/ndt/gfv128 26040904

[B313] Song H. J. Stevens C. F. Gage F. H. (2002). Neural stem cells from adult hippocampus develop essential properties of functional CNS neurons. Nat. Neurosci. 5, 438–445. 10.1038/nn844 11953752

[B314] Song J. Zhong C. Bonaguidi M. A. Sun G. J. Hsu D. Gu Y.. (2012). Neuronal circuitry mechanism regulating adult quiescent neural stem-cell fate decision. Nature 489, 150–154. 10.1038/nature11306 22842902 PMC3438284

[B315] Spassky N. Merkle F. T. Flames N. Tramontin A. D. Garcia-Verdugo J. M. Alvarez-Buylla A. (2005). Adult ependymal cells are postmitotic and are derived from radial glial cells during embryogenesis. J. Neurosci. 25, 10–18. 10.1523/JNEUROSCI.1108-04.2005 15634762 PMC6725217

[B316] Spradling A. Drummond-Barbosa D. Kai T. (2001). Stem cells find their niche. Nature 414, 98–104. 10.1038/35102160 11689954

[B317] Steiner B. Wolf S. Kempermann G. (2006). Adult neurogenesis and neurodegenerative disease. Regen. Med. 1, 15–28. 10.2217/17460751.1.1.15 17465817

[B318] Street J. M. Barran P. E. Mackay C. L. Weidt S. Balmforth C. Walsh T. S.. (2012). Identification and proteomic profiling of exosomes in human cerebrospinal fluid. J. Transl. Med. 10:5. 10.1186/1479-5876-10-5 22221959 PMC3275480

[B319] Su P. Zhang J. Zhao F. Aschner M. Chen J. Luo W. (2014). The interaction between microglia and neural stem/precursor cells. Brain Res. Bull. 109, 32–38. 10.1016/j.brainresbull.2014.09.005 25245208

[B320] Sun Y. Luo Z. M. Guo X. M. Su D. F. Liu X. (2015). An updated role of microRNA-124 in central nervous system disorders: a review. Front. Cell. Neurosci. 9:193. 10.3389/fncel.2015.00193 26041995 PMC4438253

[B321] Suntres Z. Smith M. Momen-Heravi F. Hu J. Zhang X. Wu Y. (2013). Therapeutic uses of exosomes. Intech Exosomes Microvesicles 1:56522 10.5772/56522

[B322] Szajnik M. Derbis M. Lach M. Patalas P. Michalak M. Drzewiecka H.. (2013). Exosomes in Plasma of Patients with Ovarian Carcinoma: potential biomarkers of tumor progression and response to therapy. Gynecol. Obstet. (Sunnyvale) 4:3. 10.4172/2161-0932.s4-003 24466501 PMC3899646

[B323] Takeuchi T. Suzuki M. Fujikake N. Popiel H. A. Kikuchi H. Futaki S.. (2015). Intercellular chaperone transmission via exosomes contributes to maintenance of protein homeostasis at the organismal level. Proc. Natl. Acad. Sci. U S A 112, E2497–2506. 10.1073/pnas.1412651112 25918398 PMC4434695

[B324] Tamboli I. Y. Barth E. Christian L. Siepmann M. Kumar S. Singh S.. (2010). Statins promote the degradation of extracellular amyloid β-peptide by microglia via stimulation of exosome-associated insulin-degrading enzyme (IDE) secretion. J. Biol. Chem. 285, 37405–37414. 10.1074/jbc.M110.149468 20876579 PMC2988346

[B325] Tan K. H. Tan S. S. Sze S. K. Lee W. K. Ng M. J. Lim S. K. (2014). Plasma biomarker discovery in preeclampsia using a novel differential isolation technology for circulating extracellular vesicles. Am. J. Obstet. Gynecol. 211, 380.e1–380.e13. 10.1016/j.ajog.2014.03.038 24657793

[B326] Tashiro A. Sandler V. M. Toni N. Zhao C. Gage F. H. (2006). NMDA-receptor-mediated, cell-specific integration of new neurons in adult dentate gyrus. Nature 442, 929–933. 10.1038/nature05028 16906136

[B327] Tavazoie M. Van der Veken L. Silva-Vargas V. Louissaint M. Colonna L. Zaidi B.. (2008). A specialized vascular niche for adult neural stem cells. Cell Stem Cell 3, 279–288. 10.1016/j.stem.2008.07.025 18786415 PMC6864413

[B328] Taylor A. R. Robinson M. B. Gifondorwa D. J. Tytell M. Milligan C. E. (2007). Regulation of heat shock protein 70 release in astrocytes: role of signaling kinases. Dev. Neurobiol. 67, 1815–1829. 10.1002/dneu.20559 17701989

[B329] Théry C. Boussac M. Véron P. Ricciardi-Castagnoli P. Raposo G. Garin J.. (2001). Proteomic analysis of dendritic cell-derived exosomes: a secreted subcellular compartment distinct from apoptotic vesicles. J. Immunol. 166, 7309–7318. 10.4049/jimmunol.166.12.7309 11390481

[B330] Théry C. Ostrowski M. Segura E. (2009). Membrane vesicles as conveyors of immune responses. Nat. Rev. Immunol. 9, 581–593. 10.1038/nri2567 19498381

[B331] Thompson C. A. Purushothaman A. Ramani V. C. Vlodavsky I. Sanderson R. D. (2013). Heparanase regulates secretion, composition and function of tumor cell-derived exosomes. J. Biol. Chem. 288, 10093–10099. 10.1074/jbc.C112.444562 23430739 PMC3617250

[B332] Tickner J. A. Urquhart A. J. Stephenson S. A. Richard D. J. O’Byrne K. J. (2014). Functions and therapeutic roles of exosomes in cancer. Front. Oncol. 4:127. 10.3389/fonc.2014.00127 24904836 PMC4034415

[B333] Tong C. K. Chen J. Cebrián-Silla A. Mirzadeh Z. Obernier K. Guinto C. D.. (2014). Axonal control of the adult neural stem cell niche. Cell Stem Cell 14, 500–511. 10.1016/j.stem.2014.01.014 24561083 PMC4080817

[B334] Torreggiani E. Perut F. Roncuzzi L. Zini N. Baglio S. R. Baldini N. (2014). Exosomes: novel effectors of human platelet lysate activity. Eur. Cell. Mater. 28, 137–151; discussion 151. 25241964 10.22203/ecm.v028a11

[B335] Town T. Breunig J. J. Sarkisian M. R. Spilianakis C. Ayoub A. E. Liu X.. (2008). The stumpy gene is required for mammalian ciliogenesis. Proc. Natl. Acad. Sci. U S A 105, 2853–2858. 10.1073/pnas.0712385105 18287022 PMC2268549

[B336] Trajkovic K. Hsu C. Chiantia S. Rajendran L. Wenzel D. Wieland F.. (2008). Ceramide triggers budding of exosome vesicles into multivesicular endosomes. Science 319, 1244–1247. 10.1126/science.1153124 18309083

[B337] Tran T. H. Mattheolabakis G. Aldawsari H. Amiji M. (2015). Exosomes as nanocarriers for immunotherapy of cancer and inflammatory diseases. Clin. Immunol. 160, 46–58. 10.1016/j.clim.2015.03.021 25842185

[B338] Tsilioni I. Panagiotidou S. Theoharides T. C. (2014). Exosomes in neurologic and psychiatric disorders. Clin. Ther. 36, 882–888. 10.1016/j.clinthera.2014.05.005 24913029

[B339] Turola E. Furlan R. Bianco F. Matteoli M. Verderio C. (2012). Microglial microvesicle secretion and intercellular signaling. Front. Physiol. 3:149. 10.3389/fphys.2012.00149 22661954 PMC3357554

[B340] Udo H. Yoshida Y. Kino T. Ohnuki K. Mizunoya W. Mukuda T.. (2008). Enhanced adult neurogenesis and angiogenesis and altered affective behaviors in mice overexpressing vascular endothelial growth factor 120. J. Neurosci. 28, 14522–14536. 10.1523/JNEUROSCI.3673-08.2008 19118187 PMC6671242

[B341] Ueki T. Tanaka M. Yamashita K. Mikawa S. Qiu Z. Maragakis N. J.. (2003). A novel secretory factor, Neurogenesin-1, provides neurogenic environmental cues for neural stem cells in the adult hippocampus. J. Neurosci. 23, 11732–11740. 14684875 10.1523/JNEUROSCI.23-37-11732.2003 PMC6740945

[B342] Ung T. H. Madsen H. J. Hellwinkel J. E. Lencioni A. M. Graner M. W. (2014). Exosome proteomics reveals transcriptional regulator proteins with potential to mediate downstream pathways. Cancer Sci. 105, 1384–1392. 10.1111/cas.12534 25220623 PMC4454399

[B343] Van Aelst L. N. Heymans S. (2013). MicroRNAs as biomarkers for ischemic heart disease. J. Cardiovasc. Transl. Res. 6, 458–470. 10.1007/s12265-013-9466-z 23716129

[B344] van Balkom B. W. de Jong O. G. Smits M. Brummelman J. den Ouden K. de Bree P. M.. (2013). Endothelial cells require miR-214 to secrete exosomes that suppress senescence and induce angiogenesis in human and mouse endothelial cells. Blood 121, 3997–4006. 10.1182/blood-2013-02-478925 23532734

[B345] van Balkom B. W. Eisele A. S. Pegtel D. M. Bervoets S. Verhaar M. C. (2015). Quantitative and qualitative analysis of small RNAs in human endothelial cells and exosomes provides insights into localized RNA processing, degradation and sorting. J. Extracell. Vesicles 4:26760. 10.3402/jev.v4.26760 26027894 PMC4450249

[B346] van Praag H. Christie B. R. Sejnowski T. J. Gage F. H. (1999a). Running enhances neurogenesis, learning and long-term potentiation in mice. Proc. Natl. Acad. Sci. U S A 96, 13427–13431. 10.1073/pnas.96.23.13427 10557337 PMC23964

[B347] van Praag H. Kempermann G. Gage F. H. (1999b). Running increases cell proliferation and neurogenesis in the adult mouse dentate gyrus. Nat. Neurosci. 2, 266–270. 10.1038/6368 10195220

[B348] Veena J. Rao B. S. Srikumar B. N. (2011). Regulation of adult neurogenesis in the hippocampus by stress, acetylcholine and dopamine. J. Nat. Sci. Biol. Med. 2, 26–37. 10.4103/0976-9668.82312 22470231 PMC3312696

[B349] Vella L. J. Sharples R. A. Lawson V. A. Masters C. L. Cappai R. Hill A. F. (2007). Packaging of prions into exosomes is associated with a novel pathway of PrP processing. J. Pathol. 211, 582–590. 10.1002/path.2145 17334982

[B350] Villarroya-Beltri C. Baixauli F. Gutiérrez-Vázquez C. Sánchez-Madrid F. Mittelbrunn M. (2014). Sorting it out: regulation of exosome loading. Semin. Cancer Biol. 28, 3–13. 10.1016/j.semcancer.2014.04.009 24769058 PMC4640178

[B351] Von Bartheld C. S. Altick A. L. (2011). Multivesicular bodies in neurons: distribution, protein content and trafficking functions. Prog. Neurobiol. 93, 313–340. 10.1016/j.pneurobio.2011.01.003 21216273 PMC3055956

[B352] Vukovic J. Colditz M. J. Blackmore D. G. Ruitenberg M. J. Bartlett P. F. (2012). Microglia modulate hippocampal neural precursor activity in response to exercise and aging. J. Neurosci. 32, 6435–6443. 10.1523/JNEUROSCI.5925-11.2012 22573666 PMC6621117

[B353] Wagner C. Bátiz L. F. Rodríguez S. Jiménez A. J. Páez P. Tome M.. (2003). Cellular mechanisms involved in the stenosis and obliteration of the cerebral aqueduct of hyh mutant mice developing congenital hydrocephalus. J. Neuropathol. Exp. Neurol. 62, 1019–1040. 14575238 10.1093/jnen/62.10.1019

[B354] Walton N. M. Sutter B. M. Laywell E. D. Levkoff L. H. Kearns S. M. Marshall G. P.. (2006). Microglia instruct subventricular zone neurogenesis. Glia 54, 815–825. 10.1002/glia.20419 16977605

[B357] Wang S. Cesca F. Loers G. Schweizer M. Buck F. Benfenati F.. (2011). Synapsin I is an oligomannose-carrying glycoprotein, acts as an oligomannose-binding lectin and promotes neurite outgrowth and neuronal survival when released via glia-derived exosomes. J. Neurosci. 31, 7275–7290. 10.1523/JNEUROSCI.6476-10.2011 21593312 PMC6622588

[B355] Wang G. Dinkins M. He Q. Zhu G. Poirier C. Campbell A.. (2012). Astrocytes secrete exosomes enriched with proapoptotic ceramide and prostate apoptosis response 4 (PAR-4): potential mechanism of apoptosis induction in Alzheimer disease (AD). J. Biol. Chem. 287, 21384–21395. 10.1074/jbc.M112.340513 22532571 PMC3375560

[B356] Wang H. Warner-Schmidt J. Varela S. Enikolopov G. Greengard P. Flajolet M. (2015). Norbin ablation results in defective adult hippocampal neurogenesis and depressive-like behavior in mice. Proc. Natl. Acad. Sci. U S A 112, 9745–9750. 10.1073/pnas.1510291112 26195764 PMC4534263

[B358] Wendler F. Bota-Rabassedas N. Franch-Marro X. (2013). Cancer becomes wasteful: emerging roles of exosomes(dagger) in cell-fate determination. J. Extracell. Vesicles 2:22390. 10.3402/jev.v2i0.22390 24223259 PMC3823269

[B359] Wilhelmsson U. Faiz M. de Pablo Y. Sjöqvist M. Andersson D. Widestrand A.. (2012). Astrocytes negatively regulate neurogenesis through the Jagged1-mediated Notch pathway. Stem Cells 30, 2320–2329. 10.1002/stem.1196 22887872

[B361] Winner B. Kohl Z. Gage F. H. (2011). Neurodegenerative disease and adult neurogenesis. Eur. J. Neurosci. 33, 1139–1151. 10.1111/j.1460-9568.2011.07613.x 21395858

[B360] Winner B. Winkler J. (2015). Adult neurogenesis in neurodegenerative diseases. Cold Spring Harb. Perspect. Biol. 7:a021287. 10.1101/cshperspect.a021287 25833845 PMC4382734

[B362] Woodbury M. E. Freilich R. W. Cheng C. J. Asai H. Ikezu S. Boucher J. D.. (2015). miR-155 is essential for inflammation-induced hippocampal neurogenic dysfunction. J. Neurosci. 35, 9764–9781. 10.1523/JNEUROSCI.4790-14.2015 26134658 PMC4571507

[B363] Wu A. Ying Z. Gomez-Pinilla F. (2008). Docosahexaenoic acid dietary supplementation enhances the effects of exercise on synaptic plasticity and cognition. Neuroscience 155, 751–759. 10.1016/j.neuroscience.2008.05.061 18620024 PMC3208643

[B364] Würdinger T. Tannous B. A. Saydam O. Skog J. Grau S. Soutschek J.. (2008). miR-296 regulates growth factor receptor overexpression in angiogenic endothelial cells. Cancer Cell 14, 382–393. 10.1016/j.ccr.2008.10.005 18977327 PMC2597164

[B365] Xiang J. Yan S. Li S. H. Li X. J. (2015). Postnatal loss of hap1 reduces hippocampal neurogenesis and causes adult depressive-like behavior in mice. PLoS Genet. 11:e1005175. 10.1371/journal.pgen.1005175 25875952 PMC4398408

[B366] Xin H. Li Y. Cui Y. Yang J. J. Zhang Z. G. Chopp M. (2013a). Systemic administration of exosomes released from mesenchymal stromal cells promote functional recovery and neurovascular plasticity after stroke in rats. J. Cereb. Blood Flow Metab. 33, 1711–1715. 10.1038/jcbfm.2013.152 23963371 PMC3824189

[B367] Xin H. Li Y. Liu Z. Wang X. Shang X. Cui Y.. (2013b). MiR-133b promotes neural plasticity and functional recovery after treatment of stroke with multipotent mesenchymal stromal cells in rats via transfer of exosome-enriched extracellular particles. Stem Cells 31, 2737–2746. 10.1002/stem.1409 23630198 PMC3788061

[B368] Yamamoto S. Niida S. Azuma E. Yanagibashi T. Muramatsu M. Huang T. T.. (2015). Inflammation-induced endothelial cell-derived extracellular vesicles modulate the cellular status of pericytes. Sci. Rep. 5:8505. 10.1038/srep08505 25687367 PMC4330530

[B369] Yang P. Arnold S. A. Habas A. Hetman M. Hagg T. (2008). Ciliary neurotrophic factor mediates dopamine D2 receptor-induced CNS neurogenesis in adult mice. J. Neurosci. 28, 2231–2241. 10.1523/JNEUROSCI.3574-07.2008 18305256 PMC6671859

[B370] Young S. Z. Lafourcade C. A. Platel J. C. Lin T. V. Bordey A. (2014). GABAergic striatal neurons project dendrites and axons into the postnatal subventricular zone leading to calcium activity. Front. Cell. Neurosci. 8:10. 10.3389/fncel.2014.00010 24478632 PMC3904109

[B371] Zappaterra M. W. Lehtinen M. K. (2012). The cerebrospinal fluid: regulator of neurogenesis, behavior and beyond. Cell. Mol. Life Sci 69, 2863–2878. 10.1007/s00018-012-0957-x 22415326 PMC3856656

[B372] Zhang J. Li S. Li L. Li M. Guo C. Yao J.. (2015a). Exosome and exosomal microRNA: trafficking, sorting and function. Genomics Proteomics Bioinformatics 13, 17–24. 10.1016/j.gpb.2015.02.001 25724326 PMC4411500

[B373] Zhang P. Y. Yang Y. J. Xue Y. Fu J. Zhang C. X. Wang Y.. (2015b). Cancer stem cells: targeting tumors at the source. Eur. Rev. Med. Pharmacol. Sci. 19, 1821–1828. 26044226

[B374] Zhang Y. Chopp M. Meng Y. Katakowski M. Xin H. Mahmood A.. (2015c). Effect of exosomes derived from multipluripotent mesenchymal stromal cells on functional recovery and neurovascular plasticity in rats after traumatic brain injury. J. Neurosurg. 122, 856–867. 10.3171/2014.11.JNS14770 25594326 PMC4382456

[B376] Zhao C. Sun G. Li S. Lang M. F. Yang S. Li W.. (2010). MicroRNA let-7b regulates neural stem cell proliferation and differentiation by targeting nuclear receptor TLX signaling. Proc. Natl. Acad. Sci. U S A 107, 1876–1881. 10.1073/pnas.0908750107 20133835 PMC2836616

[B375] Zhao C. Sun G. Li S. Shi Y. (2009). A feedback regulatory loop involving microRNA-9 and nuclear receptor TLX in neural stem cell fate determination. Nat. Struct. Mol. Biol. 16, 365–371. 10.1038/nsmb.1576 19330006 PMC2667220

[B377] Zhuang X. Xiang X. Grizzle W. Sun D. Zhang S. Axtell R. C.. (2011). Treatment of brain inflammatory diseases by delivering exosome encapsulated anti-inflammatory drugs from the nasal region to the brain. Mol. Ther. 19, 1769–1779. 10.1038/mt.2011.164 21915101 PMC3188748

